# Facial Image expression recognition and prediction system

**DOI:** 10.1038/s41598-024-79146-z

**Published:** 2024-11-12

**Authors:** Animesh Talukder, Surath Ghosh

**Affiliations:** Department of Mathematics, SAS, Vellore Institute of Technology, Chennai, 600127 Tamilnadu India

**Keywords:** Convolutional neural network, Image Processing, Pattern Recognition, Facial expression analysis, Classification, Signs and symptoms, Mathematics and computing

## Abstract

Facial expression recognition system is an advanced technology that allows machines to recognize human emotions based on their facial expressions. In order to develop a robust prediction model, this research work proposes three distinct architectural models to produce a facial expression prediction system that looks like this: The first model is on using a support vector machine to carry out a classification task. As a follow-up to the second model, an attempt was made to create a Convolution Neural Network (CNN) using the VGG-NET (Visual Geometry Group Network). Following analysis of the results, an attempt was made to enhance the outcome using the third model, which used convolutional sequential layers linked to seven distinct expressions, and an inference was drawn based on loss and accuracy metric behavior. We will use a dataset of human picture facial images in this research, which has more than 35500 facial photographs and represents seven different types of facial expressions. We will analyze our data and make every effort to remove as much noise as we can before feeding that information to our model. We use the confusion matrix to assess the model’s performance after it has been implemented effectively. To demonstrate the effectiveness of our model architecture, we will generate bar graphs and scatter plots for each model to display model loss and accuracy. The output of this model is visualized with actual class and predictive class and the result has a graphical representation for each and every output facial Images which makes our recognition system user-friendly.

## Introduction

Face Recognition is a type of biological method that can accurately analyze and evaluate facial patterns on facial contours to uniquely identify and verify a person. Face recognition has become a widely used and widely recognized biometric and has grown notably in significance in security-related areas. In terms of the least expensive and most commonly used touch-less biometrics, it has gained more significance during pandemic situations. In this article, we examine the effects of dimensionality reduction on the effectiveness or precision of facial recognition algorithms using machine learning. The research is conducted using a variety of algorithms, including K-Nearest Neighbor, Support Vector Machine, Linear Regression, and Logistic Regression^1–5^. Based on the experiment’s methodology, Logistic Regression works better in terms of accuracy.

Using multi-layer neural network models like CNN, deeper learning and its algorithms are one of the most recent and important branches in face recognition algorithms that aid in solving various problems with images and texts^6^. The primary challenge is to discover a more precise answer to the face tracking issue, which calls for a variety of statistical methods like factorial design and linear algebra to identify and represent eigen faces. Finally, in order to increase the accuracy of our findings, we have to acknowledge confounding variables that affect the model’s output by meddling with dependent and independent variables^7^. Every face has distinguishable landmarks, numerous, valleys and the different peaks. These are the landmarks which defines nodal points of face. Approximately 80 nodal points are there for each human face. Some of these calculated by the proposed model^8^:

- Gapping between the eyes.
- Width of the eye sockets.
- The shape of the cheekbones.
- The length of the jawline.

In our study, we capture human faces with various expressions using a photography format, leveraging online platforms like Google, GitHub, and Kaggle to compile a comprehensive dataset. Data preprocessing involves analyzing the dataset, removing blank images with pixel values of 0, and performing dimensionality reduction with Principal Component Analysis (PCA) using $n=3$ for effective visualization. We implemented three architectural models for facial recognition: the first model employs a Support Vector Machine (SVM) for classifying images into seven distinct classes, while the second and third models utilize Convolutional Neural Networks (CNNs), with the latter incorporating a sequential CNN architecture and hypothesis testing to enhance accuracy. Model evaluation is conducted using metrics such as accuracy, precision, recall, and F1 score, validated by a confusion matrix, and visualizations include accuracy plots, violin plots, bar plots, and heat maps to illustrate the impact of various features on model performance.

One of the most interesting areas of study in the field of computer vision is the detection of patterns in pictures using classifiers^9–11^. There are many real-world uses for face recognition, and recent research even leads one to believe that any specialized detectors can be presented by applying fast detection classifiers. So, we have created an algorithm that, by combining the effects of computer vision ideas, will accurately identify faces in input images. This algorithm makes use of the ideas of identifying skin tone, spotting edges, and extracting various characteristics from faces. The statistics derived from computing the parameters defining the components of the face provide proof for the finding. In this study, we’re trying to predict and recognize human facial expressions using seven different facial attitudes, including surprise, happiness, anger, disgust, fear, and happiness. Our primary goal is to identify the six types of facial expressions used by humans, including those for anger, disgust, fear, happiness, sorrow, neutrality, and surprise. To do this, we employ a range of deep learning and machine learning models, such as convolution neural networks, support vector machines, and principal component analyses. Additionally, to enhance the accuracy of our predictions, we are employing statistical techniques like factorial designs, hypothesis testing, and test statistics in^8,12,13^. As a result, we are better able to anticipate facial emotions.

## Related work

Here are some key papers and articles that have contributed to the field of facial expression recognition according to our reference:

Li, Yang and Sangwhan Cha proposed a work^1^on face recognition system about facial recognition expression using artificial neural network to operational model containing of a large number of nodes (or neurons) linked to each other. They suggested developing a face recognition system that is high performing, scalable, flexible, and reasonably priced. We break down the suggested strategy into a number of tiny side projects. We looked at neural networks and convolutional neural networks first. We constructed the Siamese network, which would train the neural network based on similarities, based on the idea of deep learning. Following a review and comparison of the open-source data sets available, the author’s selected the ORL dataset and GPU-trained the model. For data processing linear discriminant analysis takes a major role for a huge size of dataset like facial image dataset. The research work^2^done by Hafez, Samir F., Mazen M. Selim, and Hala H. Zayed expressed how we can deal with 2D face image features utilizing subset of non-correlated and Orthogonal Gabor Filters without taking the whole Gabor Filter Bank and compressing the output feature vector applying Linear Discriminant Analysis (LDA). Gu, Fuqiang, et al described about how to use a basic linear algebra approach like principal component analysis to create a crude face recognition system in^3^. Also, in anther work^4^helped us to understand about development of face recognition field, more advanced techniques are proposed, which might outperform the algorithms that we have already included in the framework. Except machine learning algorithm and convolution neural network, generative adversarial network (GAN) very powerful tool for facial expression system. Here, Gan related research work in^5^tell us about Automatic detection of GAN-faces is of emerging needs, so numerous detection approaches have been developed to combat the malicious use of GAN-faces. Physical-based approaches check for artifacts or discrepancies between the face and the real environment, including perspective lighting and reflections, to identify GAN-faces. Confounding factor which leads dependent property of each variable. A larger goal of similarly defeating all HID technologies is described in^7^. The face is divided up into zones that are modeled separately, and the output of the individual zone models are combined according to weights generated by a probabilistic likelihood model. For global face identity we need a self-learning recognition system which is a application of artificial intelligence. As an example, it is concluded in^8^that in verification, an image is suited to only one image in the database (1:1). As for an example, an image is taken of a subject may be matched to an image in the Department of Motor Vehicles database to verify the subject is who he says he is. If identification is the goal, then the image is compared to all images in the database resulting in a score for each potential match (1:N). The work^13^concluded that classification of face recognition based on real conditions needs non-ideal condition which is assign as PIE problem. To extract the model with the help of KNN classifier. In that paper main approach is to find the ideal transformation function so as to achieve the optimal recognition effect for the search process includes ANN, KNN, LDA and their applications^6^. proposed an approach that used unsupervised data that is extensively available using self-supervised learning (SSL) algorithms to recognize the emotion. The outcomes indicated that model can successfully tackle the problem of multi-modal emotion identification using the self-supervised learning (SSL) and intra-modality interaction approaches. Another work in^14^where the author did research about SoftMax functions and related application that represents a fixed inter-class angle at which optimization should stop, and it informs the optimal value of the margins for ArcFace, AmpFace, and CosFace. Namely, that angle seems to be given by the point at which intra-class distance equals half of the inter-class distance. In^15^, the proposed work is a methodology for FER by using the curve-let transform (CT) and the on-line sequential extreme learning machine (OSELM) with a radial basis function. He face is initially separated into a number of small areas known as local regions, and the CT is then applied to each local region of the face.) The major goal of doing this is to decrease the curve-let coefficients so that they can be quickly and readily categorized. In^16^, automated attendance system using facial recognition is presented. To build our database, proposed model is used with the help of Viola-Jones for face detection for further process. Also, using eigen Faces, features are extracted and using Euclidean distance as classifier, input face is recognized and finally updated the attendance sheet accordingly. The proposed model has a database with 10 users, and for each user model has taken 150 images. These 150 images of each user are used for both training and testing. And 80 In this work^9^, the proposed work discussed about Different tests are used to evaluate the suggested model implementation, such as independent testing, K-fold cross-validation, the confusion matrix, recall, precision, F1 measure, and ROC curve. The findings of this study’s calculations of the system’s sensitivity and specificity will be discussed”. Next^10^, presented results for video format data as input data which conclude that there is no correlation between the authenticity of the video and the variance in correlation scores. There does appear to be a correlation between the mean correlation scores and the authenticity of the video, where on average original videos have higher mean normalized cross correlation scores compared to the Deep-fakes. The proposed work on biologically inspired model^17^improves scene classification by integrating human gaze behavior through the unified deep active learning (UDAL) framework. Using objectness measures, local-global feature fusion, and deep gaze shifting path (GSP) learning, it detects semantically important regions. Sparsity penalties reduce redundant low-level features, and the GSP features are classified using a kernel SVM. This approach shows strong results across scenery datasets and can aid facial recognition tasks by improving focus on key facial landmarks, enhancing accuracy and robustness in various conditions^18^. This research presents a novel retinal vessel segmentation algorithm using GANs with a large receptive field. By integrating residual blocks and dilated convolutions, the method captures both fine and large-scale vessel features, achieving state-of-the-art accuracy on DRIVE and STARE datasets. Key findings include segmentation accuracies exceeding 95% and optimized deep network performance. This aligns with my ongoing CNN-based facial recognition work, where similar techniques can enhance feature extraction and improve recognition accuracy. This research^19^introduces an improved Firefly Algorithm (FA) combined with Extremal Optimization (EO), named IFA-EO, to address issues of slow convergence and local optima in standard FA. The IFA-EO algorithm incorporates three strategies: a new attraction model combining full and single attraction through a probability choice strategy, an adaptive step size based on iterations to balance exploration and exploitation, and integration of EO for enhanced local search. Experimental results on unimodal and multi-modal benchmark functions demonstrate that IFA-EO performs comparably or better than other FA and EO variants, particularly in accuracy and statistical performance. This research introduces the dConvLSTM-DCN framework for predicting vacant parking space (VPS) availability by capturing temporal and spatial correlations in historical zone-wise data. The model combines dual ConvLSTM components and a dense convolutional network, achieving high accuracy in both short-term and long-term predictions^20^. This approach can enhance our facial expression recognition task by adapting similar principles to capture dynamic changes in facial features over time.

Overall, the capacity to reliably identify and categories minor changes in facial expression is necessary for facial expression identification, which is a complicated and difficult task. However, tremendous progress has been achieved recently, and facial expression detection systems are getting more accurate and reliable thanks to the development of deep learning models and other cutting-edge methodologies.

## Problem statement

- Facial expression recognition is the task of recognizing the emotional state of a person based on their facial expressions. The goal of this challenge is to create a machine learning model that can recognize and classify various facial expressions, including happy, sad, angry, surprised, disgusted, and neutral, into distinct mood categories.
- Facial expression detection is difficult because it must contend with changes in lighting, expressions of the face, head orientation, occlusions, and individual variations in facial structure. In order to solve this issue, a large dataset of facial images must be gathered, annotated with labels showing the appropriate emotional state, and then used to train a deep learning model. The trained model can then be used to identify face expressions in live video or image feeds as well as in previously recorded video or image data.
- Mostly, influencing behavior of facial expression between 7 different facial expression between them mislead our prediction system. So, through this project we trying to remove variation factor with the help of sequential convolution neural layers for better result.

## Research methodology

For working methodology, we divide this part in 3 categories.

1. **Data Collection and preprocessing:** We use a photography format to capture human faces with various expressions. Basically, we leverage online platforms like Google, GitHub, and Kaggle. We begin by analysing the facial expression dataset, removing blank images with pixel values of 0, and then perform dimensionality reduction using Principal Component Analysis (PCA) with n=3 for effective data visualization. Also, we executed train-test split before feeding the data to the model architecture.
2. **Model Design and Selection:** We implemented three architectural models for facial recognition. The first model uses a Support Vector Machine (SVM) to classify facial images into seven distinct classes based on their features. The second and third models leverage Convolutional Neural Networks (CNNs). Model 2 utilizes a convolutional layer with a ReLU activation function to output predictions across the seven classes. Model 3 employs a sequential CNN layer with hypothesis testing aimed at improving accuracy through refined dependency checks within the neural network.
3. **Evaluation Metrics and Tools:** For model evaluation, we use standard metrics such as accuracy, precision, recall, and F1 score. To validate the classification accuracy, we employ a confusion matrix to assess performance across the seven classes. Visualizations of model predictions are created using accuracy plot, violine plot, bar plot and heat map to illustrate the influence of different features on the model’s performance.

## Dataset analysis

### Dataset description

Fer2013 dataset is a common dataset used for facial expression recognition. The dataset contains 35,887 gray-scale facial images containing 7 different emotions. We were able to locate a total of 35887 human facial expression images in this data collection. All The images are in black and white format with seven distinct expressions: ** “anger”, “disgust”, “fear”, “happiness”, “sadness”, “surprise” and “neutral”**. This dataset contains non-human images, also some improper random images which is assign as null images or noise of our dataset.

**Fig. 1:**
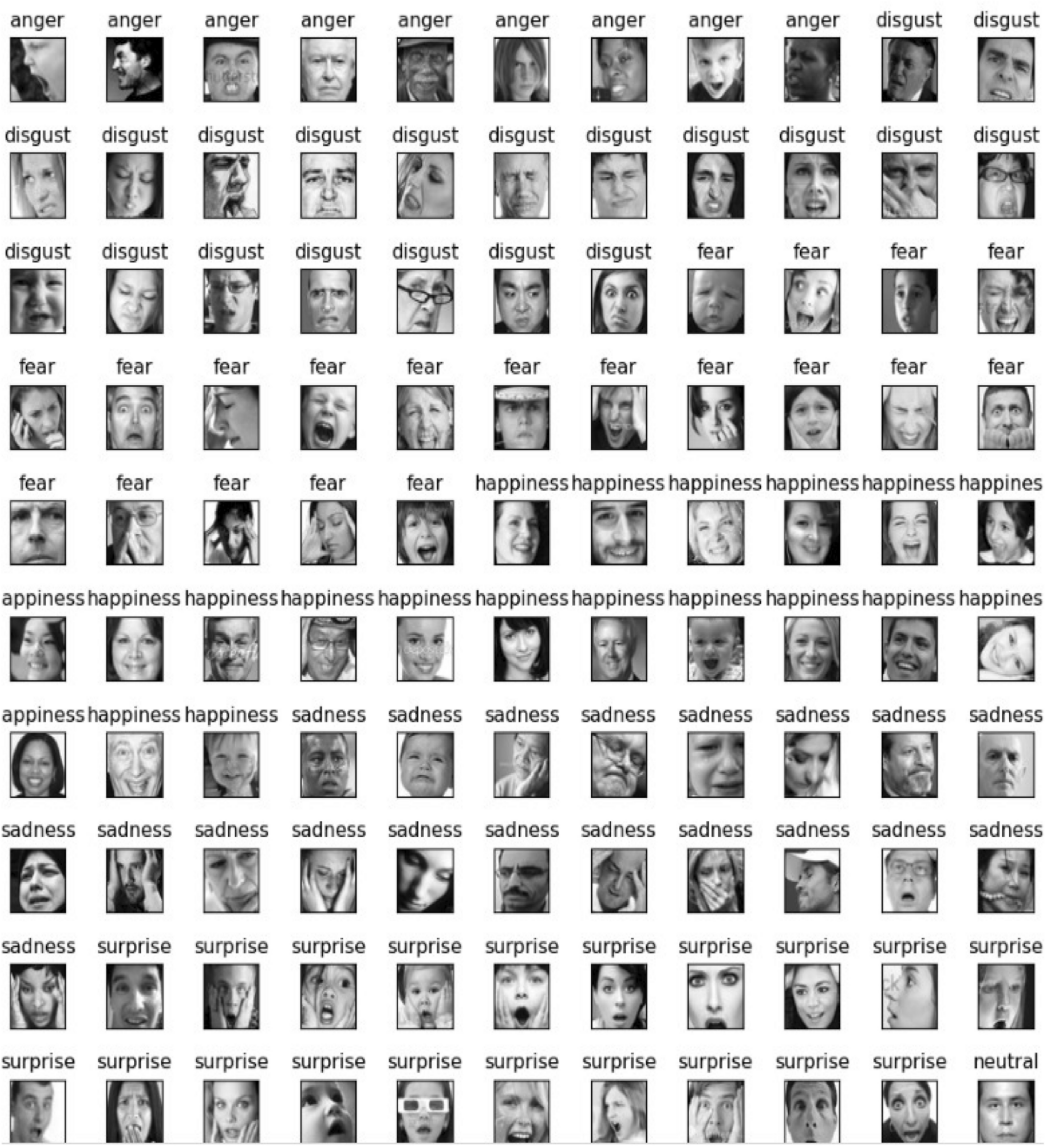
Visualization of entire dataset.

**Fig. 2:**
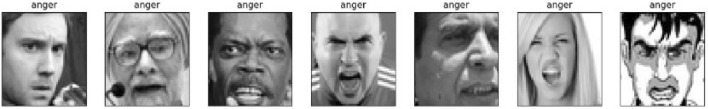
Visualization of happy dataset.

**Fig. 3:**
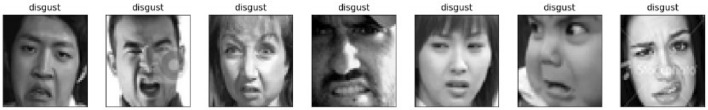
Visualization of disgust dataset.

**Fig. 4:**
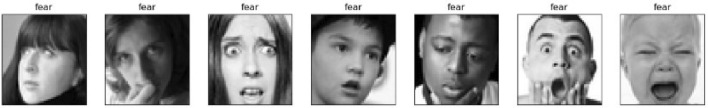
Visualization of fare dataset.

**Fig. 5:**
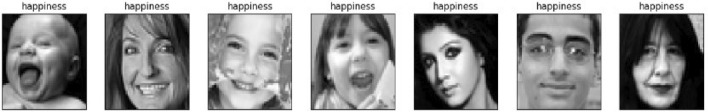
Visualization of happy dataset.

**Fig. 6:**
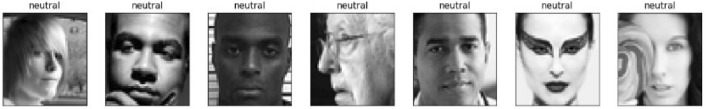
Visualization of neutral dataset.

**Fig. 7:**
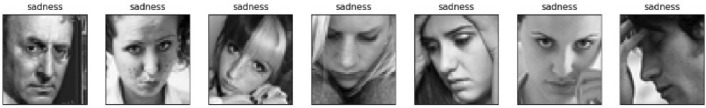
Visualization of sad dataset.

**Fig. 8:**
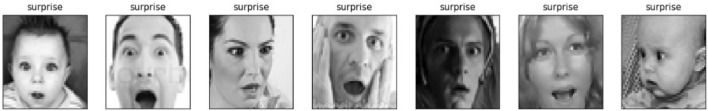
Visualization of surprise dataset.

In this Fig. 1, individual representation of each image cluster for better understanding are presented as follows :

- In the Fig. 2, there are total 4953 angry human face images. The examples are,
- According to the Fig. 3, our dataset contains total 547 disgust human face images. The examples are,
- According to the Fig. 4, our dataset contains total 5121 fare human face images. The examples are,
- Again in the Fig. 5, our dataset contains total 8989 happy human face images. The examples are,
- According to the Fig. 6, our dataset contains total 6077 neutral human face images. The examples are,
- In the Fig. 7, our dataset contains total 4002 sad human face images. The examples are,
- According to the Fig. 8, our dataset contains total 6198 surprise human face images. The examples are,

In this data set there are some misbehaved or misleading photos are there. As an example, human facial expression is sad but its assigned clustered group is angry which misleads our prediction and also, we are losing information.

### Dataset visualization

Data analysis is essential to facial recognition technology, which uses computer algorithms to analyze and recognize facial characteristics as a form of biometric identification. Even the most efficient facial recognition systems can make mistakes. Data analysis is used to locate and measure such errors and additionally to increase the algorithm’s accuracy over time. We separated the data into 7 categories for analysis. To do this, we construct a dictionary with seven groups and a range of 0 to 6 exceeding numbers that is {’Angry’: 0, ’Disgust’: 1, ’Fear’: 2, ’Happy’: 3, ’Neutral’: 4, ’Sad’: 5, ’Surprise’: 6}.

**Fig. 9:**
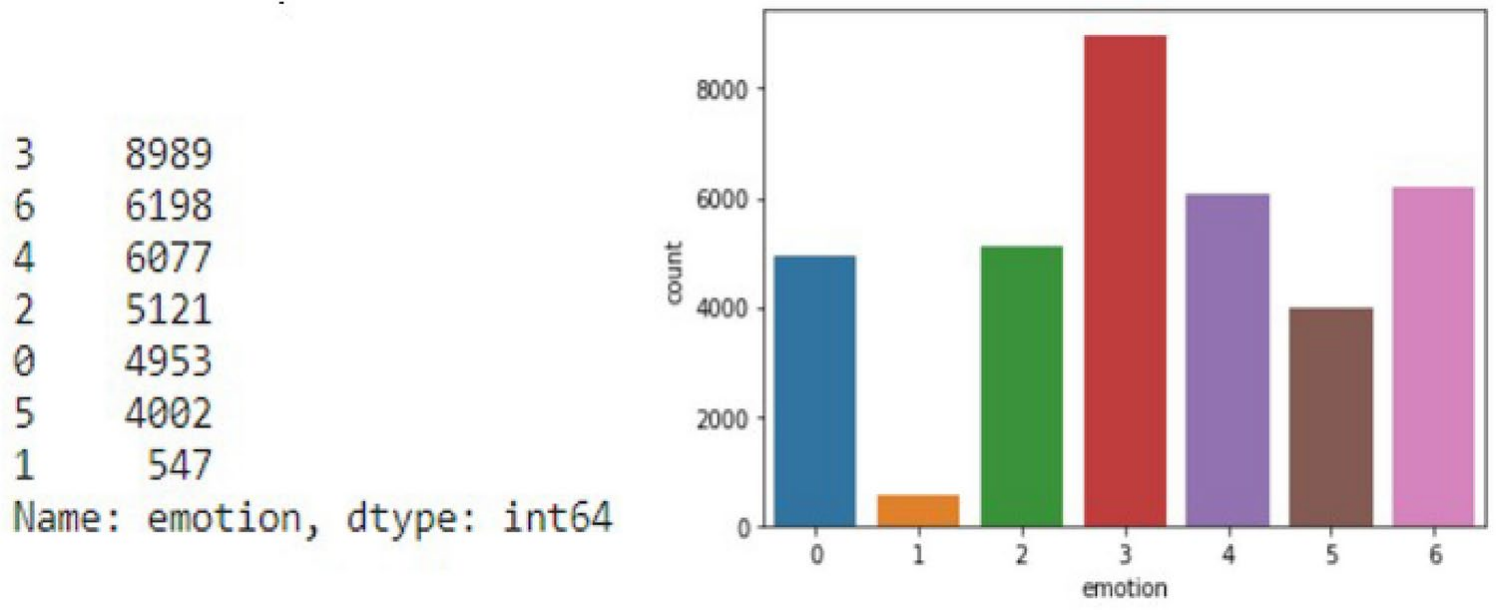
Total number of image of each attributes with bar plot.

**Fig. 10:**
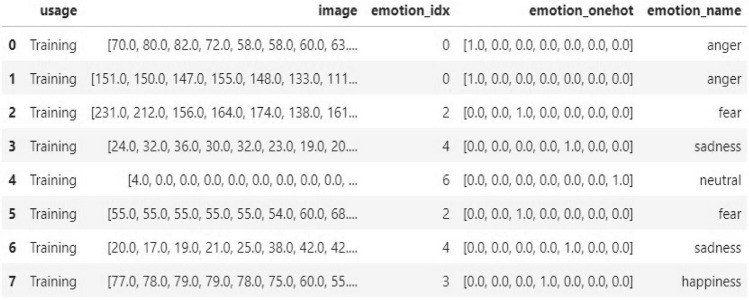
Vectorization form with pixel values.

We have total 35887 human facial images in black and white format with 7 types of different human expression. No of images with facial image data split is in the following Fig. 9,

Next, images are transformed into vector graphics through the transformation of raster images, which are made up of pixels, into vector graphics, which are made up of mathematical equations and geometric shapes. Moreover, we proceed to encode emotion numbers in a one-hot fashion. Every image is assigned in a unique category according to facial expression. We also add a column with text labels corresponding to emotion numbers. After tracing, we might need to clean up the picture by modifying the vector shapes’ anchor points and curves to guarantee accuracy and smoothness. The intensity of each pixel in a picture utilized in facial expression recognition is denoted by a number, which is commonly referred to as the pixel value. A value is given to each pixel in the image to indicate its brightness or darkness. Pixel values are used in the configuration of facial expression recognition to record the distinctive features of various facial expressions. Typically, feature extraction algorithms and other computer vision techniques are used to extract these pixel values from a picture. The intensity of each pixel in a photograph used for facial expression recognition is indicated by a number, which is commonly referred to as the pixel value. A value is assigned to each pixel in the image to reflect its brightness or darkness. Pixel values are used in the setting of facial expression recognition to record the distinctive features of various facial expressions. Typically, feature extraction algorithms along with other computer vision techniques are used to extract these pixel values from a picture. A facial expression recognition algorithm can be used to identify the individual facial expression being displayed in the picture once the pixel values have been extracted. Gray-scale and color values, such as RGB or HSV values, are frequently employed to represent pixel values for facial expressions. A data frame representation of our prepared vectorized form of individual facial image expression images as follows in the Fig. 10:

## Data pre-processing

Before feeding raw image data into a machine learning model, a number of methods are used in data pre-processing, which is additionally known as facial image recognition. Data pre-processing aims to improve the data’s quality and extract the relevant characteristics required for exact facial recognition. To make our prediction more accurate and with maximum accuracy score data processing is an important step for a good model building. For our dataset all the images are resized and in black and white format and also our data set contains properly cropped human image facial expression image dataset. In this concern we will try to split our data set into two parts which are train dataset and test dataset. Here, following dataset we followed for our data pre-processing.

### Spliting the dataset

Splitting the dataset: Our entire dataset split into two parts which are trained dataset and test dataset. Your face recognition model will be trained using the training set, and its performance on fresh, untested data can be evaluated using the test set. You might want to divide the training group again into training and validation sets. The validation set will be used to assess how well your model is working while it is being trained, while the training set will be utilized for training our model. To ensure that the data in the training, validation, and test sets are indicative of the entire dataset, make sure the split is performed at random. To do this, you can use a random number generator and the built-in features of sci-kit-learn or Tensor Flow, two machine learning frameworks. You might need to use data augmentation methods to expand your dataset because training a model for recognizing faces requires a lot of data. Make careful to only enhance the data for the training set and not the validation or test sets when doing so. Last but not the least, make sure that the data set’s distribution of classes or identities is approximately balanced across the training, validation, and test sets. In doing so, it is possible to guarantee both the validity and reliability of the evaluation findings and the training of the mathematical framework on an accurate representation of data.

**Fig. 11:**
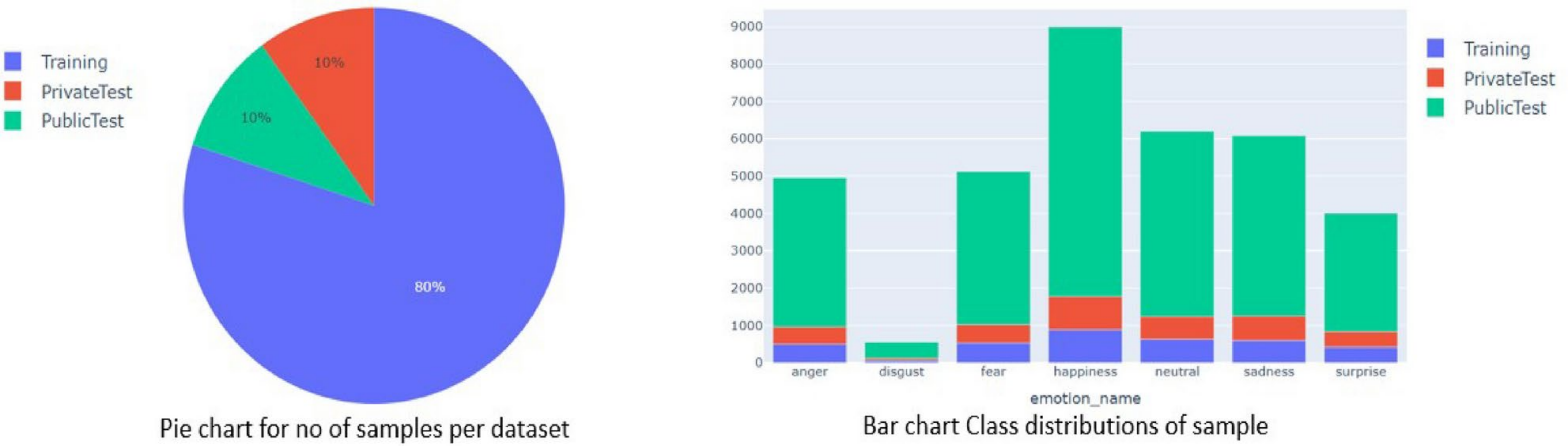
Train-Test split visualization with bar chart and pie chart.

From the pie chat Fig. 11, we are able to interpret 80% our dataset lies in training part which help us to build and learn our model architecture. For evaluate the model performance we will feed test set to our in-build model architecture and using confusion matrix we will find accuracy score, f1 score. After that, we plot accuracy plot with respect to training data and validation data and similarly loss function plot with minimized score. This pie chart shows as no of samples per dataset, on the other hand bar chart shows class distributions of sample for each individual class. In that bar chart we can conclude that the class distribution of the data is totally unbiased with respect to class distribution which help us to minimize to probability of misclassification of our model prediction and also it decreases the model loss. As a result, we get a preferable human facial recognition model with higher accuracy. As we can see all the various classes are well classified except class 1. The Disgust class is a bit under-represented. Results from the public test set are used to rank competitor’s dataset and are frequently shown on a output result. On the other hand, those taking part are kept in the dark about the confidential test set, which is only used to assess the model’s performance in its entirety. The final ranking and competition winner will be determined by considering the model’s performance on the private test set. In general, the private test set is used for final evaluation and to avoid over-fitting while the public test set is used to gouge the model’s generalizability.

### Blank images

This type of noise to make our model more efficient or else it will mislead our concerned output. This can be done manually, by visually inspecting each image and removing those that are blank or incomplete. Alternatively, automated methods such as image analysis algorithms or machine learning models can be used to identify and remove blank images from the data set. Example of improper images which are treated as blank images, It is important to note that removing blank images from a facial image data set can have implications for the accuracy and reliability of subsequent analyses. Therefore, it is important to carefully evaluate the quality and completeness of the data set before proceeding with any analyses or modeling.Some examples of assigned blank images from our dataset :

**Fig. 12:**
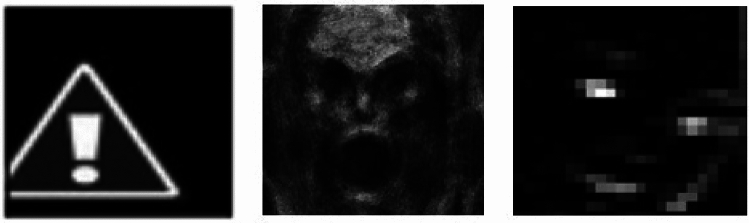
Examples of blank images.

We will find out all these type of images as Fig. 12 with a proper index number and pop-out from our training dataset. Using data frame format we can list out all this type of images and remove all. Here the table format of blank images with particular unique index number :

**Fig. 13:**
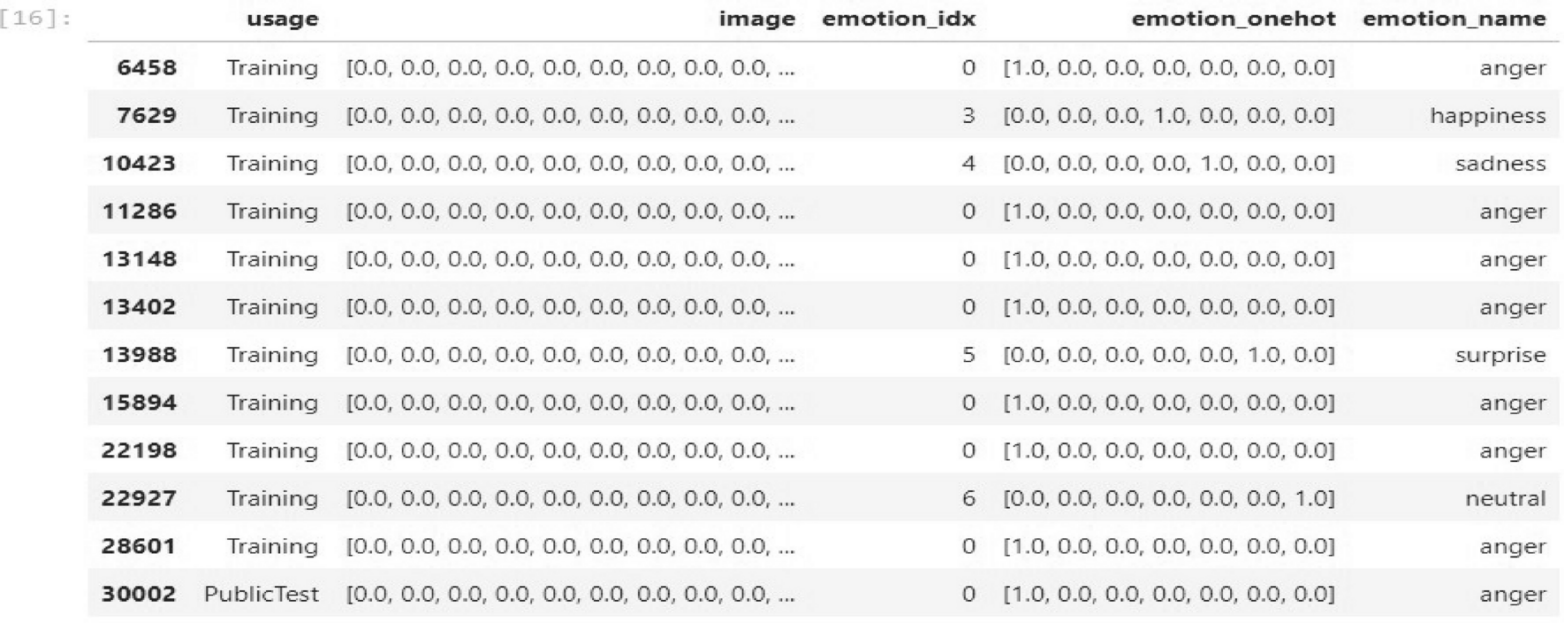
Blank images with index number.

In the Fig. 13, we can see that null images have 0 vector form for image pixel values. That means if we remove this images, it will not cause any model loss.

### Principle component analysis for dimensionality reduction

PCA is used to reduce the number of unnecessary components from our train dataset while our model still retaining most of the information. this helps us to make dataset more manageable. Using this method for reducing a data set’s complexity while keeping the most data possible. The most crucial facial features that add to the emotion being exhibited can be found using PCA in the context of recognizing human facial expressions. o use PCA for facial expression recognition, the first step is to collect a set of facial images that represent different expressions. These images should be preprocessed to ensure that they are of uniform size and are aligned properly. The next step is to extract the features from each image, which can be done using techniques such as Local Binary Patterns (LBP) or Histogram of Oriented Gradients (HOG).

**Fig. 14:**
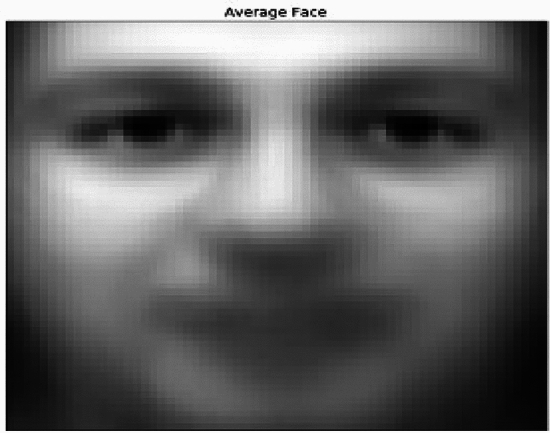
mean average face.

**Fig. 15:**
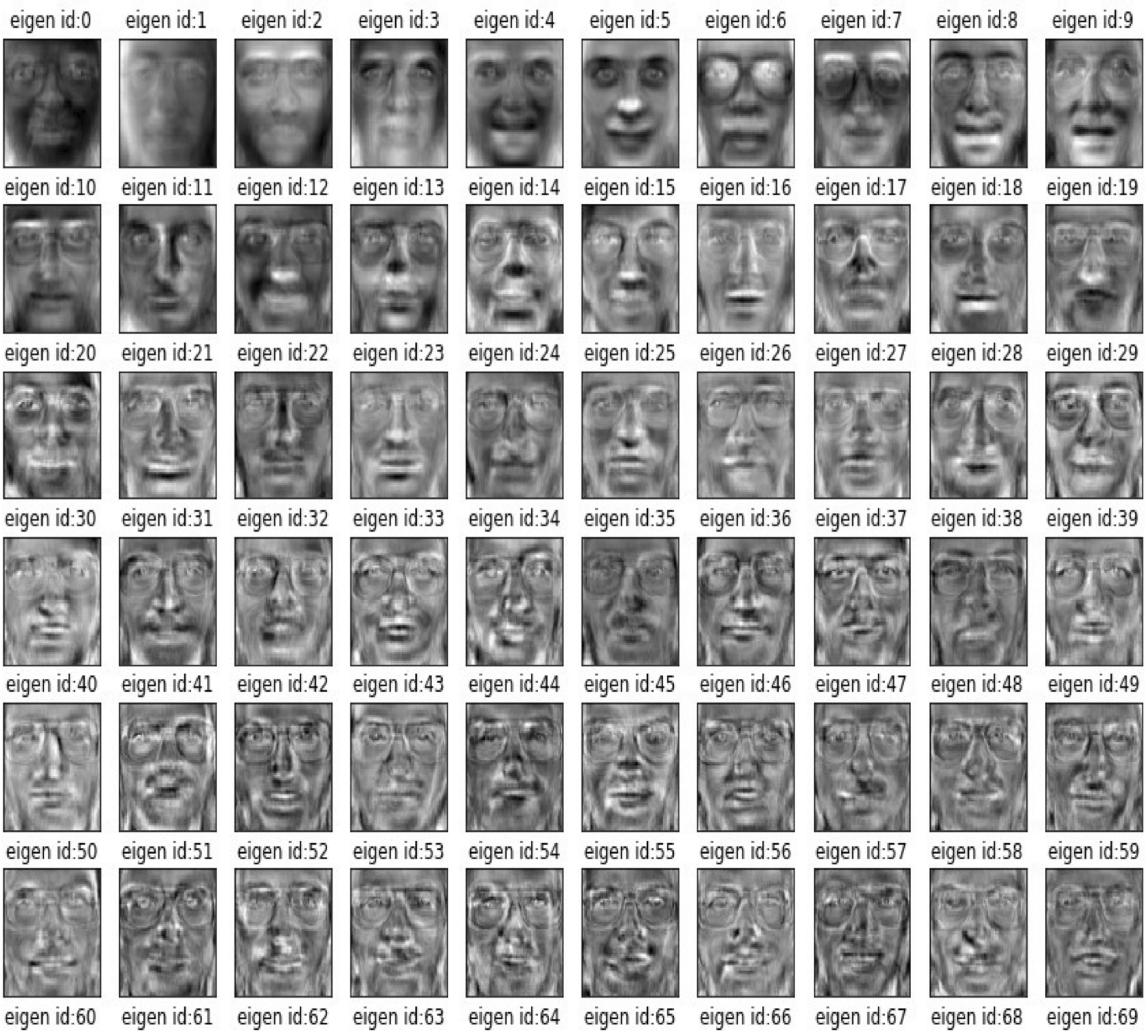
Visualization of Eigenfaces.

To implement this, we will start with mean average face. The average face, also referred to as a typical mean face, is an image that is made by adding up a number of individual facial images. The resulting image is a prototypical or average” as itself that depicts the typical facial characteristics and characteristics of the sample’s participants data. A large number of face images are aligned and normalized to make sure they are all the same size and orientation in order to produce an average mean face. In order to create an image that depicts the mean or average of all the input images, these images are then averaged together on a pixel-by-pixel basis. After this computation, the image of our mean average face of our human facial image dataset is look as follows in the Fig. 14,

It is important to note that the average mean face may not necessarily represent an ideal or attractive face, as it is derived from a statistical average of a diverse set of individuals. Additionally, the process of creating an average mean face can be sensitive to the quality and variability of the input images, and may be influenced by factors such as lighting, pose, and ethnicity. Next, using average mean face we will find covariance matrix of vectorized form facial image dataset. From covariance matrix we get eigen values which represents the individual eigen values of input human face images. Using Sci-kit learn library we trained our dataset and using covariance matrix we extract eigen values which is nothing but eigen faces which are the set of principal components that capture the most important variations in the face images. After the dimensionality of the image data has been reduced, a classifier, like a network of neurons or a support vector machine (SVM), can be trained on the reduced data to identify face expressions. The classifier gains the capacity to spot patterns in the compressed data and makes use of those patterns to categorize fresh, new pictures. In their capacity to decrease the dimensionality of the facial image data while keeping the most crucial features, eigenfaces contribute to facial expression recognition systems. In order to identify facial expressions more quickly and efficiently, the system represents the pictures of faces in a lower-dimensional environment. Furthermore, eigenfaces can aid in lessening the impact of varying lighting conditions and other outside factors that may influence facial recognition systems. Here we visualize in the Fig. 15 only first 60 eigen faces with face id to identify:

After the features have been processed extracted, PCA can be used to lower the feature space’s dimensionality. This is accomplished by calculating the eigenvectors and eigenvalues of the feature vectors’ covariance matrix. The main elements of the data are represented by the eigenvectors, and the variance that is accounted for by each principal component is expressed by the eigenvalues. Techniques like the scree plot or cross-validation can be used to calculate the number of the main elements to keep. In order to identify various facial expressions, the principal components can be used as input to a classifier like a Support Vector Machine (SVM) or a Neural Network.

PCA can be a useful tool for reducing the dimensionality of facial expression data and enhancing the precision of facial expression recognition in general. However, in order to prevent over-fitting and guarantee useful generalization performance, it is crucial to carefully preprocess the data and choose the right number of principal components. In this case, we take 3 (n=3) principle components to identify the most important features which help us to reducing over-fitting. Convert the images to gray-scale and normalize the pixel values to be between 0 and 1. Also, align the faces to a common coordinate system we are taking help of pixel values which represents the characteristic value of each individual image. Perform PCA on our dataset to obtain the steps listed below:

- This 3-dimension pca plot help us to visualize performance of facial recognition by projecting a face image onto the space spanned by the eigenfaces and then measuring the distance between the projection and the eigenfaces of known individuals. The closest match is then identified as the recognized individual.
- The set of faces is a “subspace” of the set Of images.
- It is 3 dimensional.
- This is like fitting a “Hyper-Plane” to the set of faces.
- Spanned by the vectors which represents faces.
- Generalized form :
  1 $$\begin{aligned} \text {Any Face} \approx \text {Mean Face} + a_1 v_1 + a_2 v_2 + a_3 v_3 + \dots + a_k v_k \end{aligned}$$
  Where, a1 ,a2 ,a3,a4,a5,.......,ak are scalers and v1,v2,v3,v4,v5,.....vk are vectors form of corresponding eigen faces.

Here, we take n=3 that mean 3 most important principle components for each and every 7 type of human facial expression in 3 dimensional plot with 7 type of different color to identify each components individually.

**Fig. 16:**
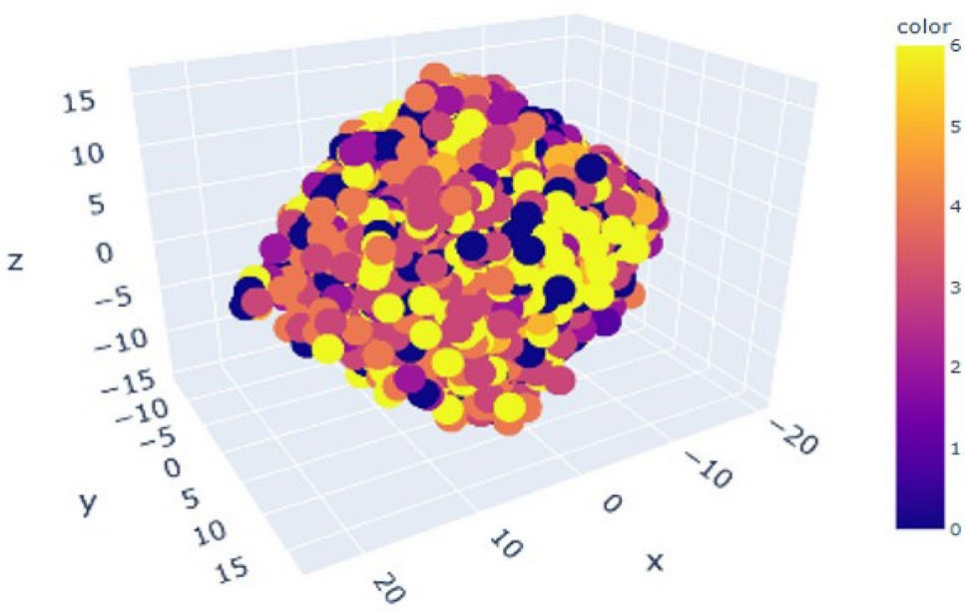
Visualization of entire dataset.

**Fig. 17:**
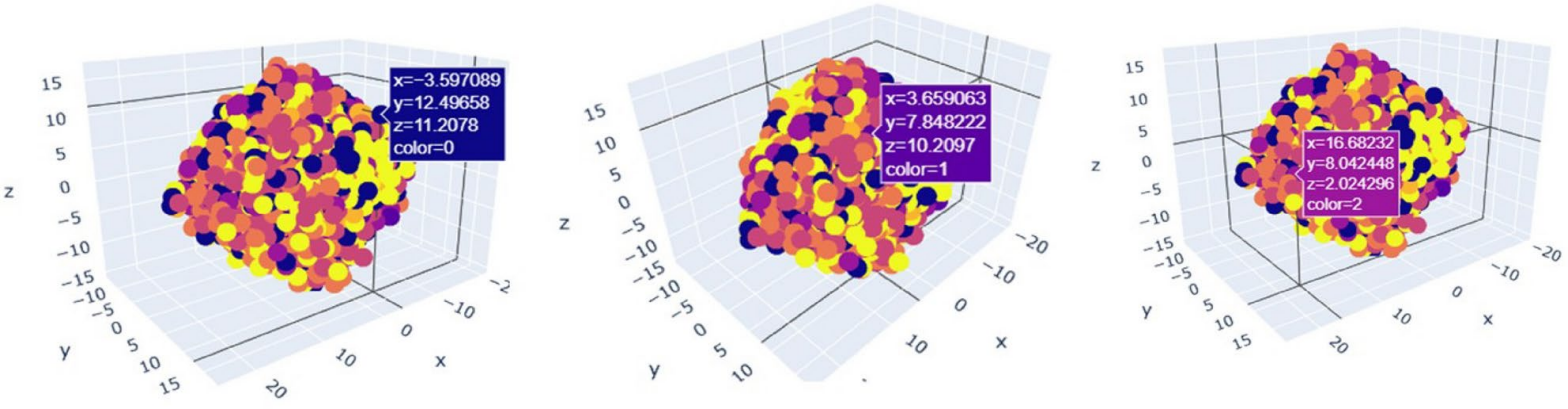
PCA plot for angry, disgust, fear.

**Fig. 18:**
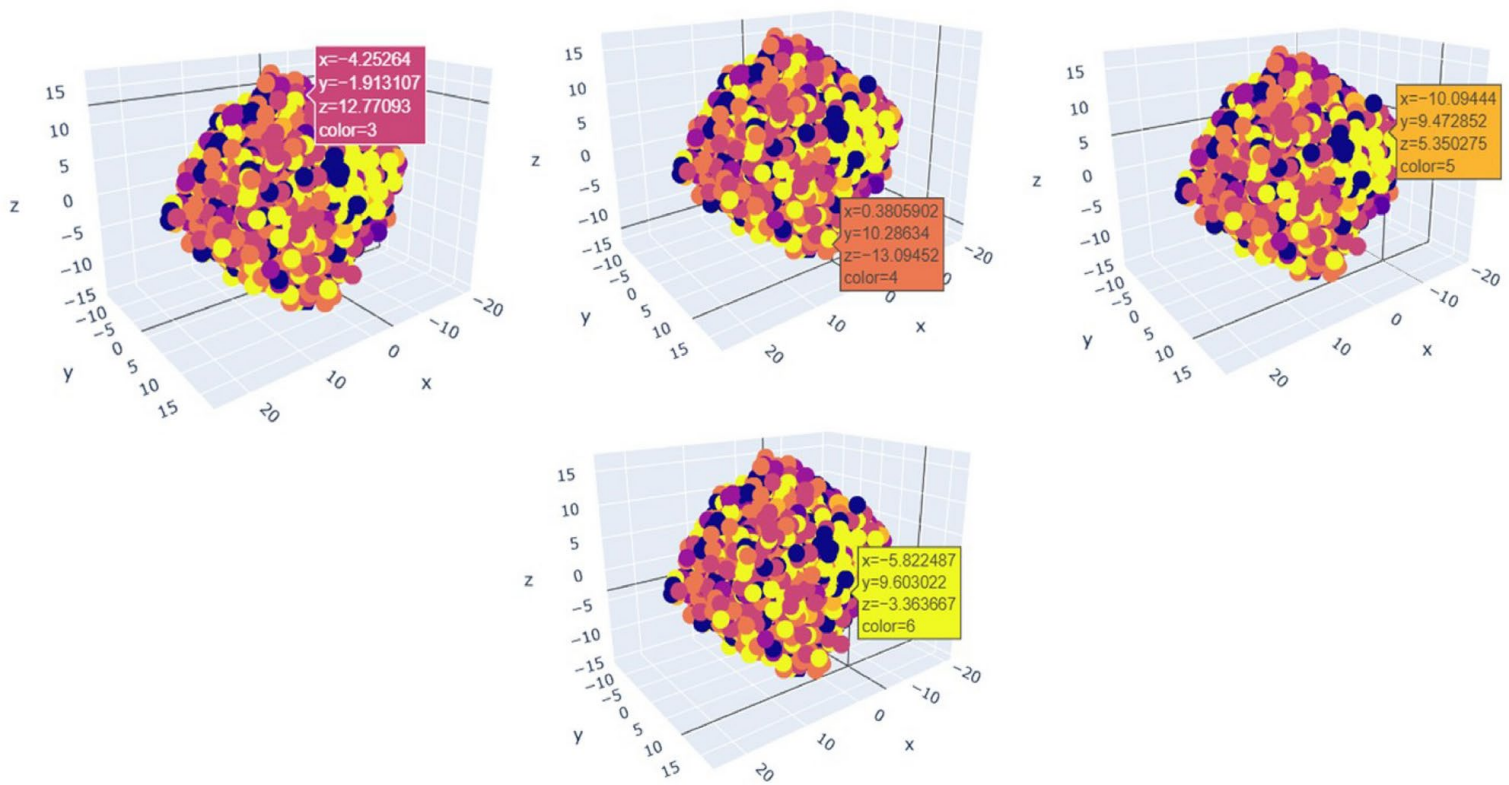
PCA plot for happy, neutral, sad, surprise.

Here, Fig. 16 represents 7 different type of principle component values with corresponding co-ordinates of our 7 different expressions with 3 dimensional scatter plot of our training dataset for each expression looks like Figs. 17 and 18.

A classifier, such as a network of neurons or a support vector machine (SVM), can be trained on the reduced data to recognize facial expressions after the dimensionality of the image data has been reduced. The classifier obtains the ability to identify patterns in the condensed data and uses those patterns to classify new, unseen images.

Feature extraction also a critical step for our image expression recognition system but this process leads to be information losses which causes our prediction wrong and make our validation data recognition performance low. In that case to extract more the most relevant features from the facial images, which can help to implement convolutional neural network in bounded by the only principal component analysis. With this step we have done data preprocessing part of our training dataset by carefully selecting and appropriate preprocessing techniques, we can believe that the system is robust, accurate and capable of accurately recognizing facial expressions in a variety of real world facial expressions.

## Model building architectures

There are several approaches to modeling the architecture for facial recognition, but here we will implement one popular method of machine learning model using support vector machine and 2 different types of convolutional neural network. For classification task, we train a classification algorithm such as support vector machine to classify the feature vector in 7 types of different classes based on their corresponding identities. For deep learning algorithm we make a convolutional neural network with two types of approach. After this step, we will evaluate our architecture model using metrics such as accuracy, precision, recall, and F1 score. To make sure that it recognizes faces from various ethnicities, ages, and genders, the structure should be trained on a diverse collection of facial images. The system should be resistant to changes in posture, lighting, and facial expressions. The system should be built with suitable security measures in place to prevent unauthorized access to the system, ensuring the privacy of the people whose faces are being identified. In this process Mostly we will concentrated to make a support vector machine and classify each expression variation in 7 different type of human facial images for that reason we use confusion matrix as a test statistics. And in the next step we will implement a convolutional neural network and our aim is to Improve accuracy score of our model and based on that we will try to make a sequential pretrained convolutional neural network model to improve prediction and make the user interface more upgraded version and try to plot our prediction space using sea-born and matplotlib libraries to show hoe one feature influencing each other. Here we conclude 3 types of architecture model and for every part we use confusion matrix to evaluate our model.Introduction of our 3 types of architecture of modes are follows :

- **Model 1**: We will implement support vector machine as classified image in 7 different classes. We will perform feature extraction after this.
- **Model 2**: Here we used a convolution layer connected to 7 different classes which outputs 7 different values corresponding to each of the 7 output feature. As activation we used Relu.
- **Model 3**: Here we used a convolution sequential layer and trying to implement hypothesis testing on dependency check of neural network and conclude a inference based on accuracy improvement.

### Implementation of model-1 architecture

By training the model on a dataset of labeled images of faces with various emotions, Support Vector Machines (SVMs) can be used to recognize facial expressions. The objective is to train the model to spot patterns and characteristics in the pictures that signify various face expressions. SVMs can effectively separate groups with a nonlinear decision limit and can handle highly dimensional feature spaces, which is one benefit of using them for expression detection. SVMs, however, can be delicate to the regularization and kernel function settings selected, which may impact the model’s success. As a result, it’s essential to choose and adjust these factors carefully. y utilizing kernel features, SVMs are capable of recognizing nonlinearly separable data. SVMs can be used to accurately model the complicated relationship between the facial features and the expression labels in expression identification, enabling the recognition of subtle expressions. Models for recognizing facial expressions may be affected by noise or changes in illumination, which may reduce their accuracy. SVMs can successfully distinguish classes even in the presence of noise or outliers because they are noise-resistant. SVMs can be used to pick the features which are most pertinent for identifying facial expressions. SVMs can convert the initial feature space into a higher-dimensional space with superior data separation by employing a kernel function. This modification may help in decreasing the dimension of a feature space and stressing important features.

Small sample size: In facial expression recognition, it may be difficult to obtain a large dataset due to the limitations of data collection. SVMs are able to perform well even with a small sample size, making them suitable for facial expression recognition models. We already done train-test split of the data, it can be deployed to perform facial recognition task. The SVM will classify each new image based on its learned model and the accuracy of the classification can be measured by comparing the predicted labels to the true labels. Support vector machines offer one way to improve on this. rather than merely drawing a zero-width line between the classes, we can bring around each edge a margin of some width, up to the nearest point.

Each image contains nearly 127 pixels. We could proceed by merely using each pixel value as a feature, but often it is more effective to use some sort of preprocessor to extract more meaningful features; here, we will use a principal component analysis to remove 67 fundamental components to feed into our support vector machine classifier.

#### Model-1 evaluation 

In multi-class classification, each class is treated as a binary classification problem against all other classes. This is often called a “one-vs-all” or “one-vs-rest” approach. We can get a better sense of our estimator’s performance using the classification report, which lists recovery statistics label by label:

**Fig. 19:**
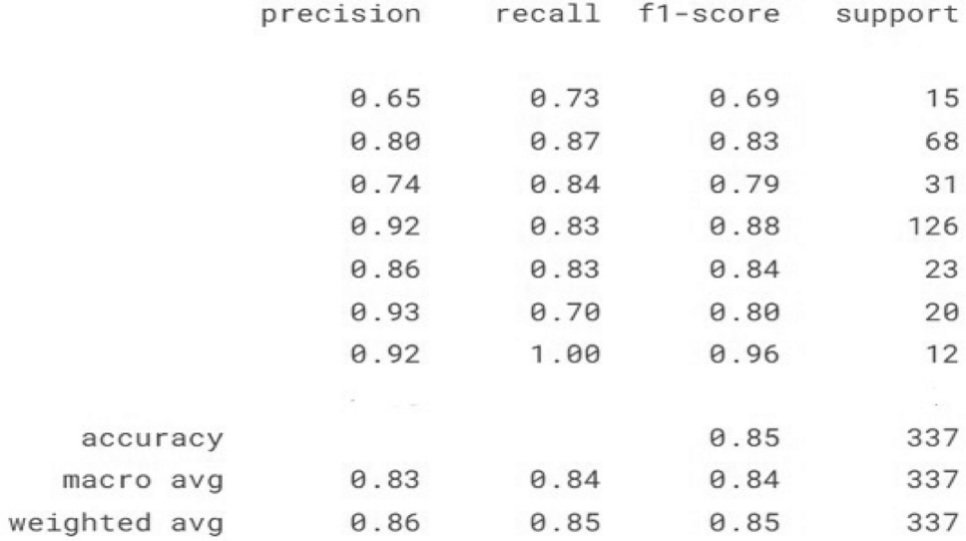
Confusion matrix for support vector machine plot.

Now with this cross-validated model, we can predict the labels for the test data. SVM is one of the older machine learning algorithms but still effective algorithm for facial recognition tasks. Here, we are dealing with 7 classification tasks with huge facial image dataset. For each class we get individual accuracy metrics (precision, Recall, F1 Score) in Fig. 19 add on with that represents the number of supports refers to the number of actual instances of each class in the dataset. As an example, first expression angry has support value of 15 which means there are 15 actual instances of this class in the test data similarly for all the others expressions which indicates our model uses only a small portion of the data was used to test the model which causes major disadvantage of SVM known as under-representation in test data. The accuracy equation is:

$$\begin{aligned} \text {Accuracy}= & \frac{\text {TP} + \text {TN}}{\text {TP} + \text {TN} + \text {FP} + \text {FN}}\\ \text {Recall}= & \frac{\text {TP}}{\text {TP} + \text {FN}}\\ \text {F1-Score}= & 2 \times \frac{\text {Precision} \times \text {Recall}}{\text {Precision} + \text {Recall}}\\ \text {Precision}= & \frac{\text {TP}}{\text {TP} + \text {FP}}\\ \text {Micro Average Precision}= & \frac{\sum _{i=1}^{N} \text {TP}_i}{\sum _{i=1}^{N} \text {TP}_i + \sum _{i=1}^{N} \text {FP}_i}\\ \text {Weighted Average Precision}= & \frac{\sum _{i=1}^{N} \text {Precision}_i \times \text {Support}_i}{\sum _{i=1}^{N} \text {Support}_i} \end{aligned}$$

where:

- TP = True Positives
- TN = True Negatives
- FP = False Positives
- FN = False Negatives

As a result, we get accuracy score is 0.85, micro average (mean of precision) is 0.83 and weighted average (Weighted mean of precision) is 0.86 with test data size of 337. Lastly selecting appropriate kernel for the project is a big issue which depends upon user’s requirement. To Address this problem, we are implementing VGG network for its ability to extract deep features from images, especially for complex tasks like the total number of parameters in the model is 20,024,384, all of which are trainable. These parameters are the weights and biases that the model learns during training.

### Implementation of model-2 architecture

We Design the CNN architecture by choosing the number of layers, the types of layers, the activation functions, and the number of filters in each layer. A typical CNN architecture for facial expression recognition may include convolutional layers, max pooling layers, fully connected layers, and dropout layers. Take advantage of an algorithm, such as stochastic gradient descent or Adam, and a loss function, such as category cross-entropy, to train the CNN on the training data. The objective is to maximize the accuracy of the model on the training data while minimizing damage. Set the CNN’s hyperparameters, such as learning rate, group size, number of filters, and number of hidden units, corresponding to the validation set. This is carried out to enhance the model’s achievement on the validation the collection. For a Convolutional Neural Network, also known as an CNN to operate at its best, it is crucial to tune the hyperparameters for face emotion recognition.

#### Building the architecture for model-2 

Here are some hyper-parameters that can be tuned:

- **Learning rate:** The learning rate controls how quickly the weights of the network are updated during training. A higher learning rate may cause the model to converge faster, but it may also cause the model to overshoot the optimal weights and result in poor performance. A lower learning rate may result in slower convergence but may lead to better performance. Less than 1.0 and larger than 106 are the range of numbers for the learning rate to be taken into account. The learning rate is typically set to 0.1 or 0.01, and this could be a suitable place to start with your issue.
- **Batch size:** The batch size determines how many images are processed at a time during each training iteration. Better convergence and generalization may result to batches that are smaller, but the training length may also rise. Larger quantities might speed up training, yet they could also result in over-fitting. The batch size for this cnn model is 128.
- **Number of filters: **The number of filters in each convolutional layer controls the number of features that are learned by the network. The ability of the model to extract complicated features can be improved by including more filters, but performing so may also raise the risk of overfitting. The VGG19 model has 19 layers and consists of 16 layers of convolutional layers and 3 layers of fully connected layers. Convolutional layers extract features from the input image using filters. The last layer of the VGG19 model consists of fully connected layers. These layers are used to classify facial expressions in the dataset. Since there are 7 different emotion classes in the Fer2013 dataset, the last layer of the VGG19 model is retrained to have a 7-output classifier.
- **Dropout rate:** During training, a particular amount of neurons are haphazardly eliminated through the dropout rate regularization method. In order to prevent over-fitting, this makes the method learn more reliable characteristics. The equilibrium between under-fitting and over-fitting can be optimized by modifying the percentage of attrition.
- **Epochs:** The complexity of the model, the size of the training dataset, and the rate of convergence during training may all influence the number of epochs is needed to build a convolutional neural network (CNN) for face expression identification. In order to achieve high accuracy, it is normal to train the model for many epochs during the course of a few hours to a few days when training a CNN for expression identification. We take 30 epochs to learn our model architecture with maximum convergence state.

**Fig. 20:**
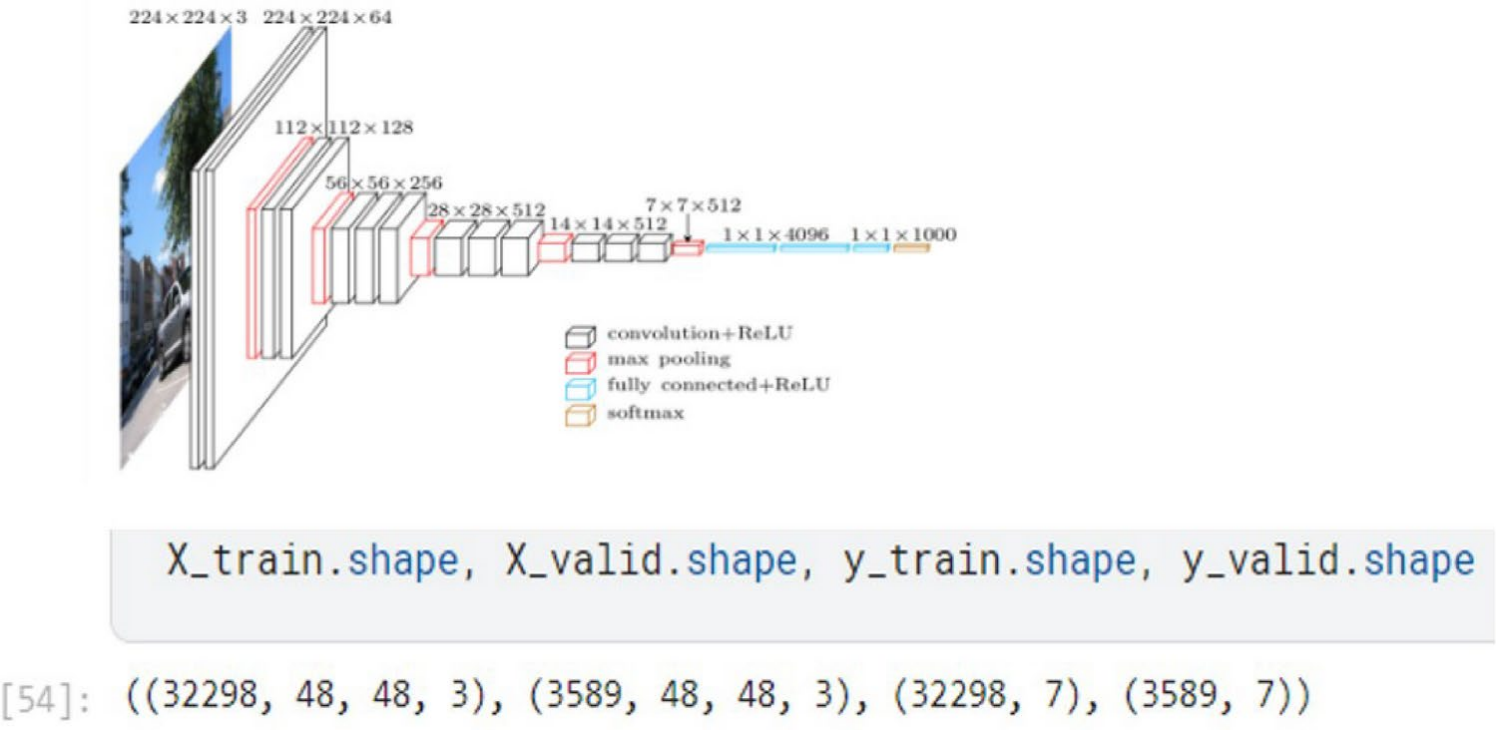
Visualization of entire dataset.

The input data reshape structure looks like Fig. 20 in dimensional shape with proper estimation for each and every layer according to the condition of our trained model with respect to trained dataset and validation dataset.

We can see that from the input data shape, total 32298 facial images are there with the shape of 48 in height and 48 in width 3 parameters. Similarly, for validation dataset we have 3589 no of photos with height of 48 and weight of 48 and 3 parameter estimation. Next we feed this data to our convolution neural layer model and trying to predict and match actual class of test dataset. Here, model 2 architecture which represents all the convolutional layers, hidden layers and total no of parameter extractions in each step-by-step process. The diagram representation is as follows :

**Fig. 21:**
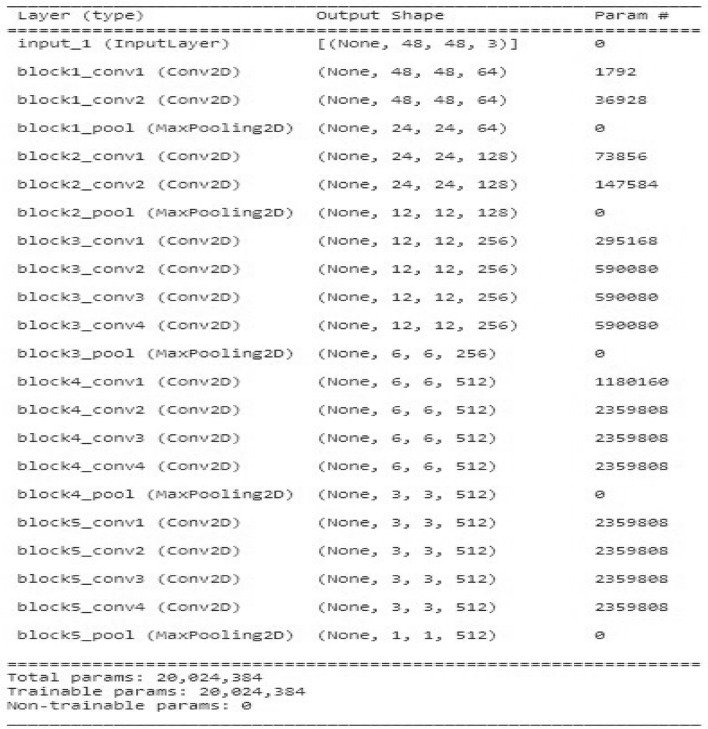
Architecture Of Convolutional Neural Network.

The summary of CNN based on the Fig. 21 is given below:

- **Input Layers and Deeper Convolutional Blocks:** The input size is (48, 48, 3), indicating that the images used in the model are RGB (3 channels) with dimensions of 48x48 pixels.The model consists of 5 convolutional blocks. Each block is made up of several convolutional layers (Conv2D) followed by a max-pooling layer. As we move deeper into the network, the number of filters increases, which allows the network to capture more complex and abstract features.Block 1 starts with 64 filters, and as we progress, the number of filters doubles, going from 128 filters in Block 2, to 256 in Block 3, and then 512 filters in both Block 4 and Block 5. Max-pooling layers after each block reduce the spatial dimensions of the image, which helps in lowering the computational load while preserving the important features learned by the convolutional layers.
- **Parameter Distribution:** The total number of parameters in the model is 20,024,384, all of which are trainable. These parameters are the weights and biases that the model learns during training.The majority of the parameters are concentrated in the deeper layers (Blocks 4 and 5). These blocks contain 512 filters, each requiring a large number of parameters due to the 3x3 convolutional kernels and the fully connected connections.Blocks 1 to 3, despite being important for learning low- and mid-level features, have far fewer parameters because they contain fewer filters.
- **Model Depth and Max Pooling:** Early layers in VGGNet capture simple features like edges, while deeper layers (Block 4 and 5) capture complex, abstract patterns needed for facial expression recognition. The deep 16-layer architecture enables the model to learn fine details but results in a large number of parameters. Max-pooling after each block reduces spatial dimensions and computational complexity, focusing on key features and enhancing translation invariance.

Moving ahead, now we analyzing the performance of our architecture model and analyzing the improvement of accuracy score and evaluating epochs in each steps and with each steps of epochs monitoring the behavior of loss function which should be a monotonic decreasing sequence and on the other hand our accuracy should be a monotonic increasing sequence with every step of learning. Let’s see numeric value table of our test statistics which are accuracy score, precision score, recall score, f1 score.The tabular form is visualize as follows :

**Fig. 22:**
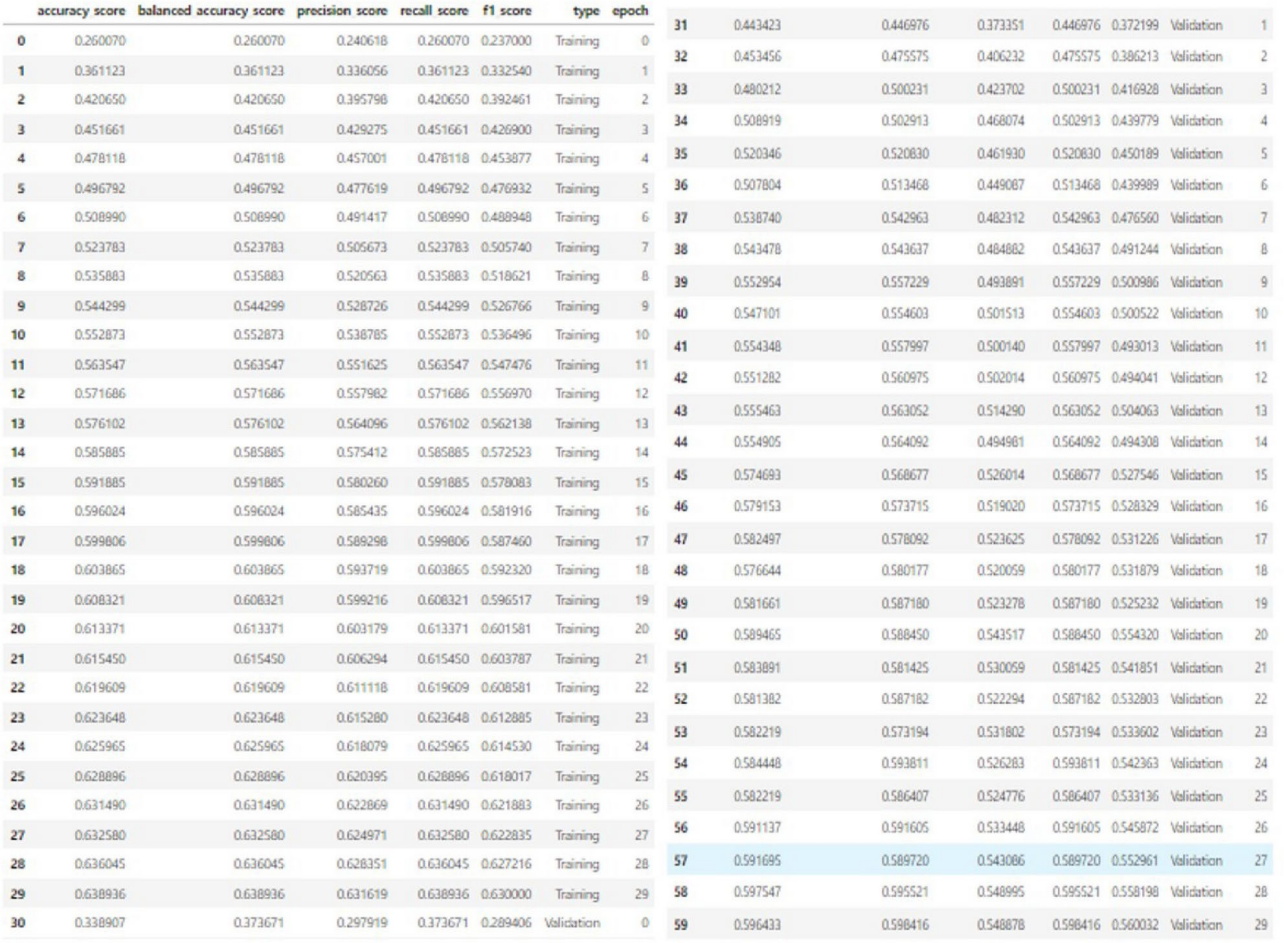
Summary Of Metric scores with corresponding epochs.

This table from Fig. 22 combinedly show as the test statistics score for both of the case trained dataset and validation dataset. In that table index number 0 to 29 we implemented 30 epochs successfully for trained dataset with accuracy score of 0.636045 which is a good score but not the best in model 3 we are trying to improve the score of our model. 0.638936 is the balanced accuracy score, precision score is 0.631619 and f1 score is 0.630000. From index no 30 to 59 total 30 epochs are situated for our validation dataset. For the validation data all the performance scores are nearby the trained data test statistics score which represent our model performance is a good position and we can trust our model. The accuracy score for validation data is 0.596433 and similarly, balanced accuracy score is 0.548878, precision score is 0.548878 and f1 score is 0.560032.

Next, we try to plot our loss function with respect to each epochs and the learning rate for this case is 0 to 0.50. Let’s see the model response against training dataset and validation dataset. This representation of loss function is in 2- dimensional format. Total 0 to 89 numerical values we show for projection of co-ordinates of loss function,

**Fig. 23:**
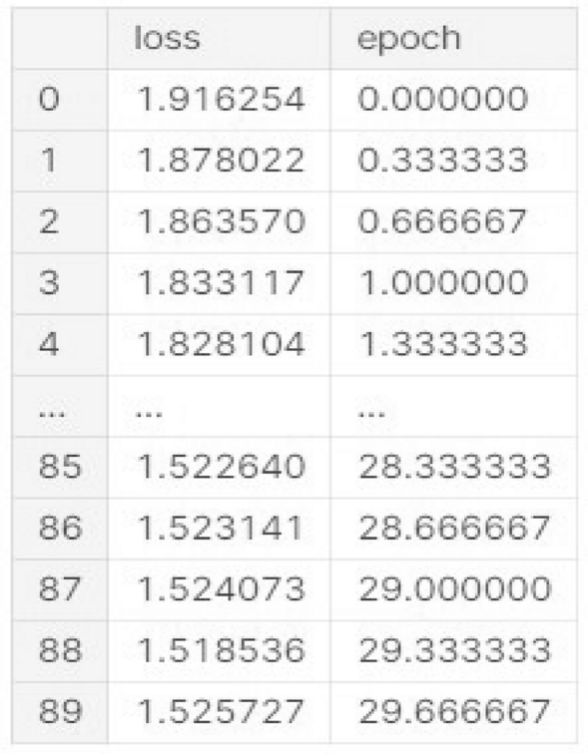
Co-Ordinates Of Loss Function.

According to the Fig. 23, the loss starts at 1.916 at epoch 0 and gradually decreases as the epochs progress, indicating the model is improving its predictions. By epoch 89 (final row), the loss value has decreased to 1.525, showing convergence. The progression in loss reduction, from 1.916 to approximately 1.525, suggests the model is learning well during training, but it appears to have reached a plateau by the end of the training period (around epoch 89), as the difference between loss values in the last few epochs is minimal. This could indicate the model has mostly converged, or additional fine-tuning is required for further improvements.

#### Model-2 performance evaluation and visualization 

Next the violine plot shows us the representation data set element spam in normal distribution format for both model validation and model trained data.The corresponding violin plot visualization is follows :

**Fig. 24:**
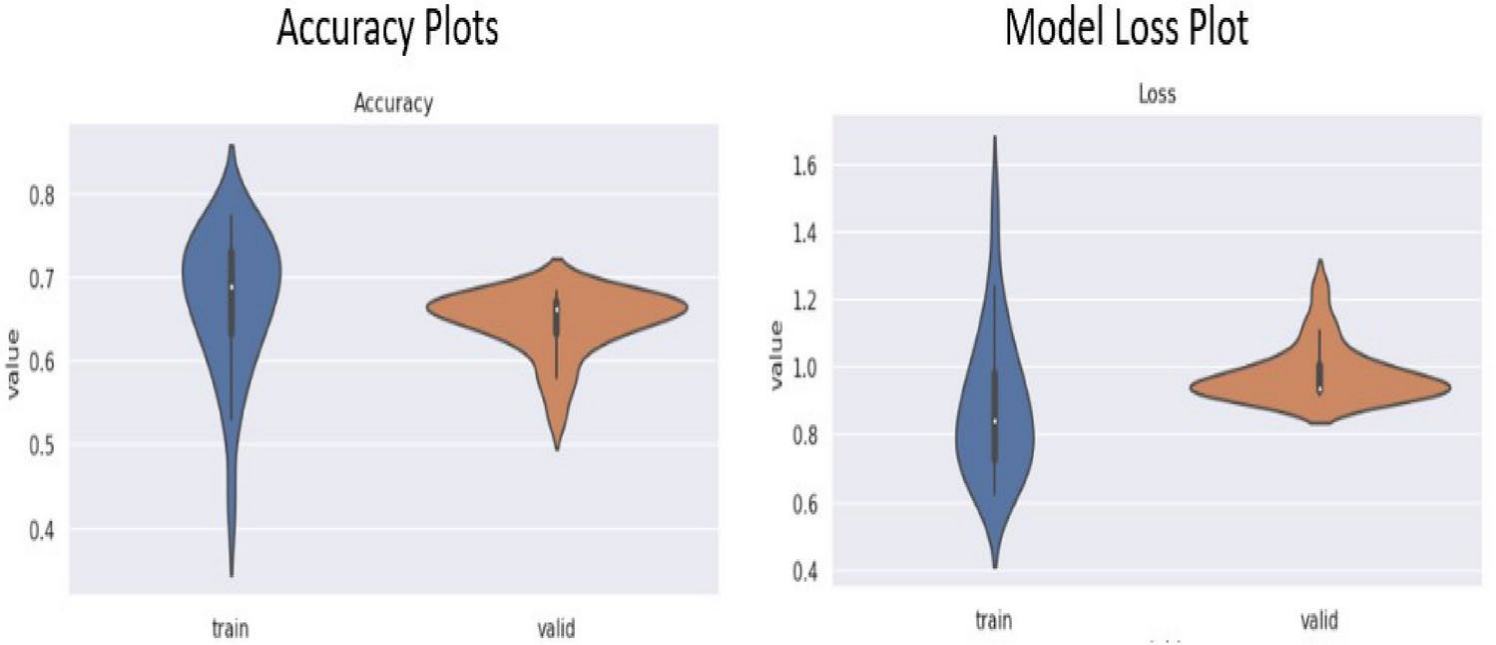
Violine plot for accuracy And loss function.

The violin plots in the Fig. 24 compare the accuracy and loss between training and validation sets for the model which shows us 1st quartile, median and 3rd quartile for both Accuracy plot and model loss plot. For the accuracy plot training set has median at 0.7 and interquartile range lies in between 0.65 to 0.75 and for validation data the interquartile range is approx 0.68 and the interquartile range is approximately lying in between 0.66 to 0.69 that means the training set has a higher concentration of values around 0.75, indicating good performance during training, while the validation accuracy centres lower, around 0.65, revealing a drop in performance on unseen data. Now for model loss we got two violin plots which are training and testing plots. For training the median is 0.82 and the interquartile range is 0.65 to 1.0. Similarly for validation data the interquartile range is 0.87 to 1.1 and the median is 0.9. Next, we plot Accuracy function and also model loss plot with to train and validation dataset. This function is plotted as accuracy plot versus on of epochs and loss plot vs no of epochs.

**Fig. 25:**
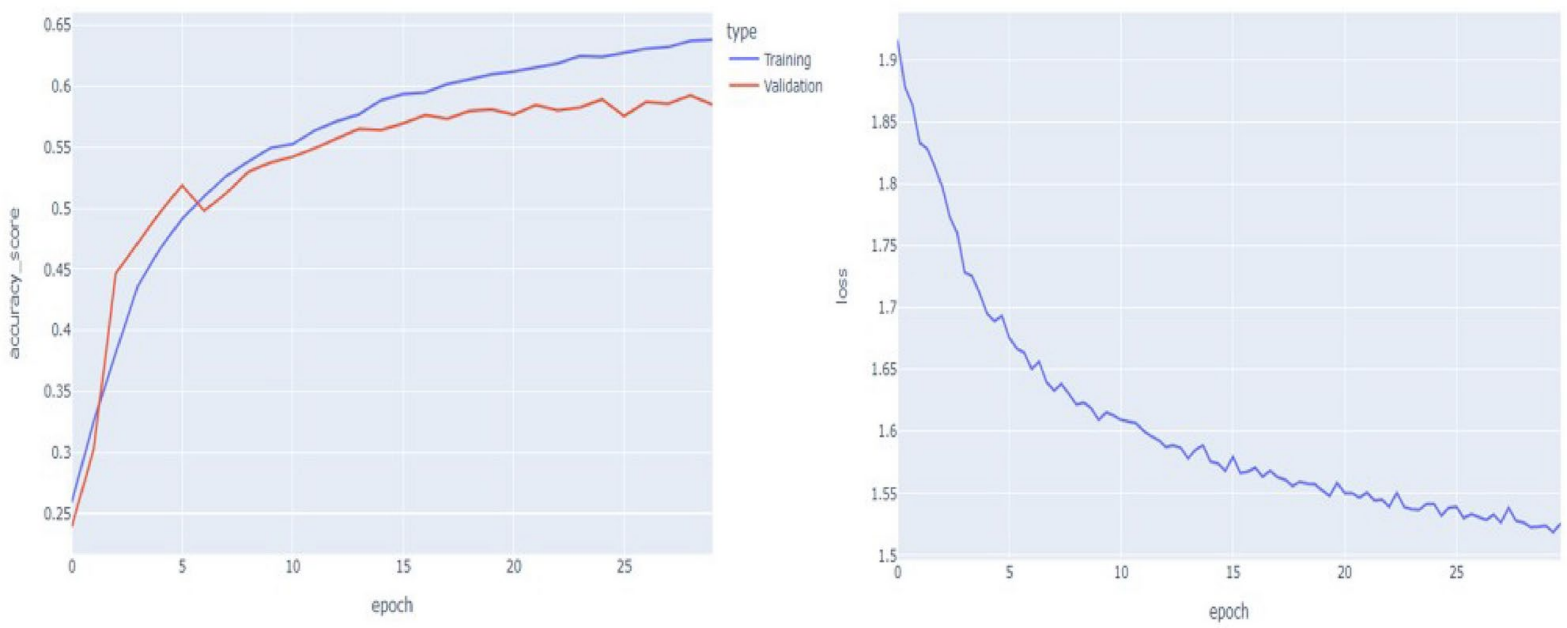
Accuracy function plot and loss function plot.

For left hand side image, we can see that with number of epochs increasing for that reason our accuracy score is monotonically increasing but our validation line is nearby of our training line in the beginning validation line shows an exponential growth but after 10 epochs it shows a linear growth which shows that our model is capable of predicting human facial expression recognition and able to classify output in 7 different types of expressions. With respect to no of epochs both lines have almost same trajectory. For right hand side we plot the model loss which represents the trained data. In this case we feed only training model loss data to evaluate our system performance against no of epochs which is 29. Training Accuracy: The accuracy during the training process is computed at each epoch based on the model’s predictions on the training dataset. The formula for each epoch is:

$$\begin{aligned} \text {Accuracy} = \frac{\text {Total Predictions}}{\text {Total Correct Predictions}} = \frac{\text {Total Values in Matrix}}{\sum \text {Diagonal Values}} \end{aligned}$$

Validation Accuracy: Similarly, the accuracy for validation is calculated based on the model’s predictions on the validation dataset:

$$\begin{aligned} \text {Validation Accuracy (epoch)} = \frac{\text {Correct Validation Predictions}}{\text {Total Validation Samples}} \end{aligned}$$

Loss Plot: The loss is minimized through the training process and plotted here. The loss is often computed using categorical cross-entropy for a multi-class classification task like facial recognition. The formula for cross-entropy loss is:

$$\begin{aligned} \text {Loss} = - \frac{1}{N} \sum _{i=1}^{N} \sum _{c=1}^{C} y_{ic} \log ({\hat{y}}_{ic}) \end{aligned}$$

Where,

- *N* is the total number of samples.
- *C* is the number of classes.
- $y_{ic}$ is the true label (1 if the sample belongs to class *c*, otherwise 0).
- ${\hat{y}}_{ic}$ is the predicted probability for class *c*.

we can easily conclude that model loss function plot is exponentially decreasing according to the Fig. 25. In the loss plot, the training loss is more concentrated, with lower values around 0.75, while the validation loss is slightly higher. As a result, we can minimize loss function as much as possible.

Next, we implement confusion matrix to evaluate model performance with 7 types of class which are angry, disgust, fear, happy, sad, neutral, surprise. But, our dataset contains human facial expression with disgust with very less number of facial expression photos, so, in prediction case our model does not able to predict disgust photos with proper way which leads some problem for accuracy score. Using heat-map representation of confusion matrix we will visualize accuracy score, f1 score, precision and recall for each of the 7 types of facial expression. Here, the confusion matrix with performance evaluation is as follows :

**Fig. 26:**
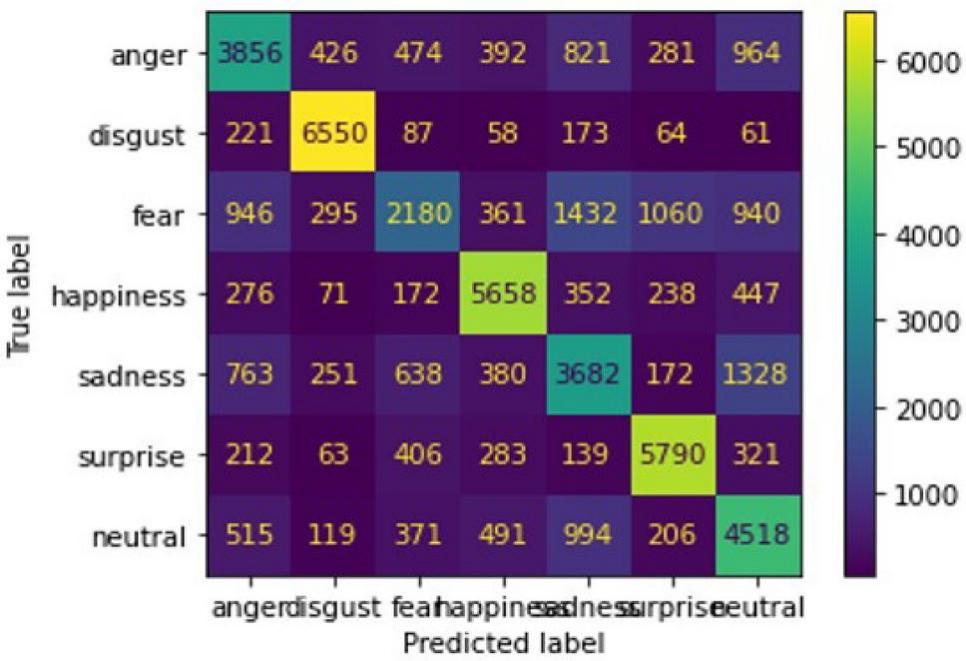
Confusion Matrix Of Training dataset.

Here is a description based on the numeric results from the confusion matrix with heatmap representation generated earlier for training data:

- **Anger:** Out of 7069 samples, the model correctly classified 3856 as “anger.” However, 946 samples were misclassified as “fear,” 964 as “neutral,” and 821 as “sadness.” There were also smaller misclassifications into other categories like “disgust” and “happiness.”
- **Disgust:** The model performs exceptionally well with this class, correctly predicting 6550 out of 7214 samples as “disgust.” Only a small number of samples are misclassified, such as 221 into “anger” and 87 into “fear.”
- **Fear:** Out of 8214 samples labeled as “fear,” the model correctly identified 2180. However, there is significant overlap with other emotions, leading to 1432 samples being misclassified as “sadness,” 1060 as “surprise,” and 946 as “anger.”
- **Happiness:** The model performs well in this category, correctly identifying 5658 out of 7214 samples as “happiness.” There are minor misclassifications, such as 447 into “neutral” and 352 into “sadness.”
- **Sadness:** Out of 7206 samples labeled “sadness,” the model correctly predicted 3682. However, there is a notable confusion with “neutral” (1328 misclassifications), as well as some confusion with “fear” and “anger.”
- **Surprise:** The model identifies 5790 out of 7214 samples as “surprise” correctly, with few misclassifications into “fear” and “neutral.”
- **Neutral:** The model predicts “neutral” well, with 4518 out of 7214 correct classifications. However, it confuses some samples with “sadness” (994 instances) and “anger” (515 instances).

This Fig. 26 represents performance of training data through the model 2 architecture. In the next step we are going to feed our validation data to our model 2 architecture. Now we will analyze the performance of our system result with similar heat-map representation of confusion matrix. data analysis for validation The performance of a face expression recognition model using conventional neural networks can be better understood thanks to confusion matrix. The number of accurate and inaccurate forecasts the model generated based on the validation information is displayed in a table called the confusion matrix.One can assess the model’s accuracy, precision, recall, and F1 score for each class of facial expressions by examining the confusion matrix. This data can be used to determine which expressions the method excels at and which ones it has issues to. Here, heat-map representation of confusion matrix for validation data is following:

**Fig. 27:**
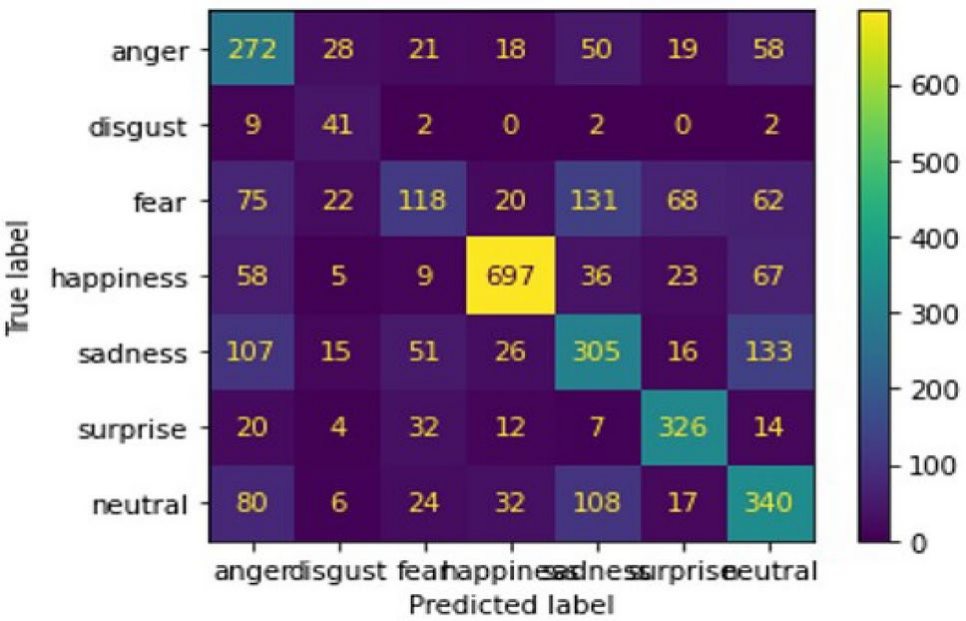
Confusion matrix Of validation dataset.

Fig. 27 is a description based on the numeric results from the confusion matrix with heatmap representation generated earlier for test set:

- **Anger:** Correctly classified in 272 cases, but some confusion with fear (75) and neutral (80) indicates that distinguishing between anger and these emotions remains challenging.
- **Disgust:** Accurately predicted in 41 instances, with minimal misclassifications. However, some confusion exists with anger (9) and sadness (15).
- **Fear:** Correctly classified 118 times but shows significant misclassification into sadness (131) and neutral (62), indicating overlapping features between fear, sadness, and neutral expressions.
- **Happiness:** Strong performance with 697 correct classifications, showing that positive emotional cues are well recognized. Some minor confusion with neutral (67) suggests possible overlap with more neutral expressions.
- **Sadness:** Out of the total, 305 samples are correctly classified, but there is a notable confusion with fear (131) and neutral (133), indicating that subtle differences between sadness and these emotions are harder to detect.
- **Surprise:** The model predicts 326 samples of surprise correctly, with minor confusion with neutral (14) and fear (32), which could be attributed to shared facial features.
- **Neutral:** Correctly identified 340 times, but misclassified into sadness (133) and anger (80), suggesting neutral expressions sometimes resemble negative emotions.

Comparison of the Training and Testing Confusion Matrices: The comparison between the training and testing confusion matrices reveals notable patterns. Anger is correctly classified 3856 times during training but only 272 in testing, with consistent confusion with fear and neutral in both sets (e.g., 946 vs. 75 for fear). Disgust shows high accuracy in training (6550) and similarly strong results in testing (41), with minimal misclassifications. Fear suffers from confusion with sadness and neutral in both cases, being correctly identified 2180 times in training but only 118 in testing. Happiness maintains robust performance, with 5658 and 697 correct classifications in training and testing, respectively. Sadness, however, continues to overlap with neutral and fear, showing 3682 correct in training and 305 in testing. Surprise is well recognized in both sets, with 5790 in training and 326 in testing. Finally, neutral struggles similarly across both sets, with 4518 in training and 340 in testing, frequently confused with sadness. This suggests the model overfits to subtle emotional distinctions in training but generalizes moderately well. The maximum accuracy score for our model 2 is 0.638936 at epoch no 29 for training data and for validation data it is 0.598745. Minimize loss score for training dataset is 1.525727 at epoch number 29.99997.The classification task performance is good as we can see from the output but it has some chance to improve toward prediction of human expression in 7 different type of domain. As we can see on the basis of our output some of the human expression images are misclassified because of their confounding behavior.

#### Output

**Fig. 28:**
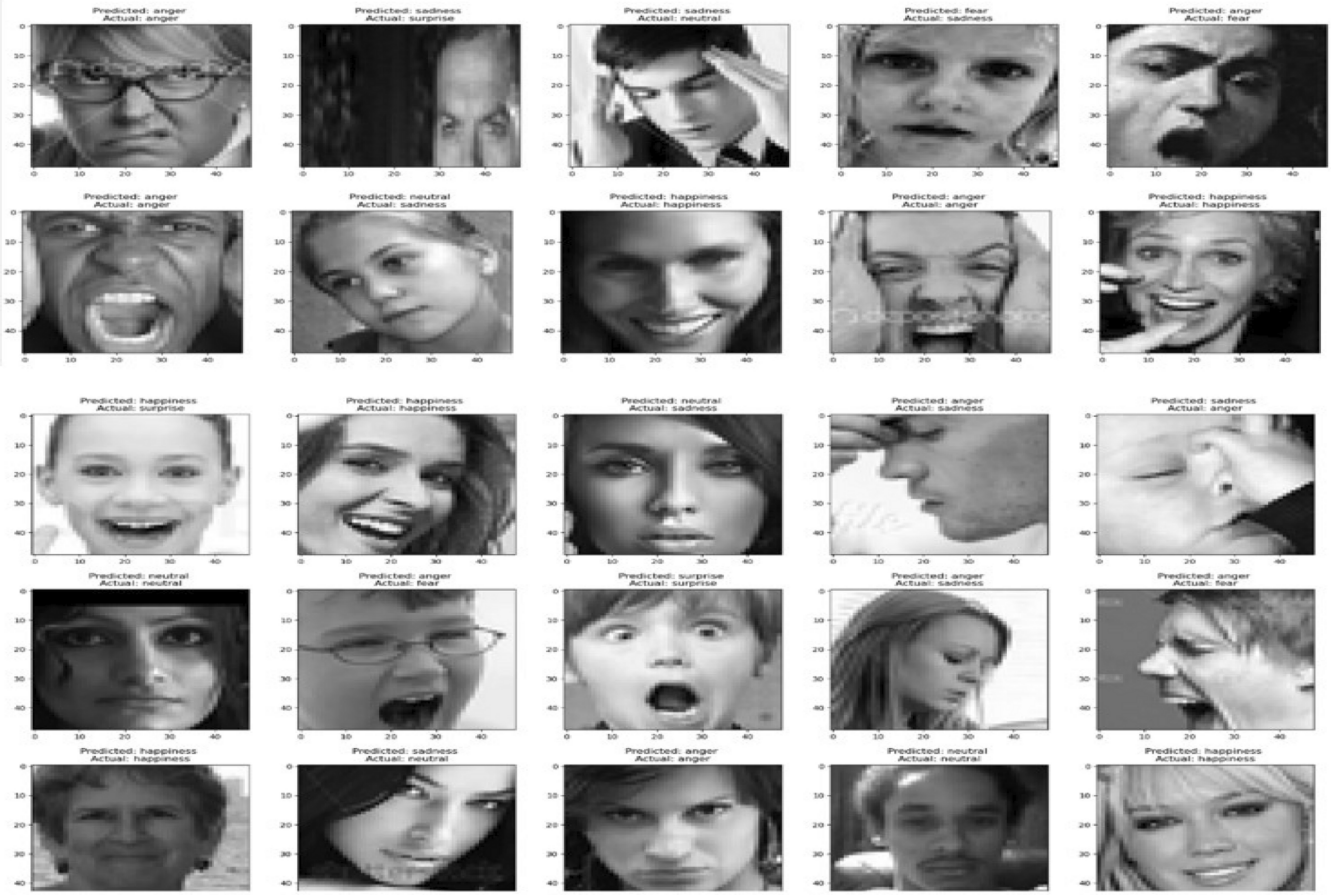
Visualization of output for facial expression model-2.

For some faces in the Fig. 28, the model correctly predicted the emotion. For instance, a few examples where “Predicted: happiness” aligns with “Actual: happiness.” There are cases of misclassification, such as “Predicted: anger” while the actual emotion is “surprise” or “fear.”The model appears to make predictions across various classes rather than favoring a particular one, suggesting that it can differentiate between most emotions to some extent, although there are clear challenges with certain emotional overlaps. Anger and sadness seem to be commonly confused. For instance, several cases labeled as sadness were predicted as anger. Neutral expressions are often mistaken for emotions like sadness or surprise. Misclassifications could be due to subtle differences in facial features or low-quality image data, which may confuse the model, especially with emotions that share visual similarities like fear and sadness. Our system has some difficulty when it tries to predict side profile of any human or face with makeup or children face with awkward position because of dependency of each other between the facial images.To achieve a more accurate answer, let’s attempt to solve this issue using a sequential fully linked CNN layer.

Our system has some difficulty when it tries to predict side profile of any human or face with makeup or children face with awkward position because of dependency of each other between the facial images.

### Implementation of model-3 architecture

#### Testing of hypothesis

In the context of convolutional neural network (CNN) layers, hypothesis testing can be used to evaluate the significance of the observed differences between the output of different layers or between the output of the same layer for different inputs. A method of statistics called hypothesis testing is used to determine, given a sample of data, whether a theory about a demographic measure is accurate or not. The significance of noted variations between the output of the same layer for various inputs or between the output of different layers can be assessed in the context of convolutional neural network, or CNN, layers via hypothesis testing. We could select a sample of images from our dataset as a collection of training dataset and feed them into the CNN layer with the goal to test the hypothesis that the output of a particular CNN layer differs for images for human face expressions compared to images of human black and white facial expressions. We could then compare the output using a statistical test such as a t-test or ANOVA. We can deny the null hypothesis and come to the conclusion that there is a difference between the way the CNN layer responds to photographs of human facial expressions if the test results show that the variance in output between the two groups is statistically significant. hypothesis testing can be a useful tool for evaluating the performance of CNN layers and for understanding how they respond to different types of inputs. It’s vital to remember that hypothesis testing relies on assumptions about the underlying statistical distribution of the data, and these presumptions may not always be true for complex information like images. Therefore, care should be taken when interpreting hypothesis test outcomes in the context of CNN layers. To compute the efficiency of our model and as evaluation of model we implement test of hypothesis between our pre-trained model and our major aim is that to improvement of prediction spaces with better accuracy score as compare to before. And also trying to reduce confounding factor from our output as a result we can able to plot the influencing probability nature between 7 different types of human facial expressions as we see before.

To compute the efficiency of our model and as evaluation of model we implement test of hypothesis between our pre-trained model and our major aim is that to improvement of prediction spaces with better accuracy score as compare to before. And also trying to reduce confounding factor from our output as a result we can able to plot the influencing probability nature between 7 different types of human facial expressions as we see before.

**Null Hypothesis:** No significant difference between the distribution of features extracted from the dataset of face images and the distribution of features extracted from a reference set of face images.

**Alternate Hypothesis:** : There is a significant difference between the distributions of features extracted from different faces, and that this difference can be used to accurately classify or identify different individuals.

Accuracy, Precision, recall is going to use as test statistics for this hypothesis testing. The null hypothesis asserts that any observed differences in the features extracted from face images are caused by random chance or noise in the data, and that the CNN model is not capable of effectively differentiating between various faces. Our method is to train the CNN model on a reference set of face images and then using it to extract features from a test set of face images with the goal to evaluate the null hypothesis in face recognition using CNNs. A statistical test, such as a t-test or an ANOVA, is then used to compare the extracted features to the features extracted from the reference set in order to ascertain whether there is a substantial difference between the two distributions. The alternative theory claims that the predicted facial expression and the input picture have a meaningful connection. It shows that the cnn model can classify various facial expressions correctly with a degree of statistical significance that is not just the result of chance.This is further explained by the fact that we train a CNN model for facial expression recognition on a dataset of labeled images that represent different facial expressions. In order to accurately forecast the facial expression, the model learns to spot patterns in the images. The alternative theory claims that the model can correctly classify new facial expression images by learning from the training data and generalizing it. We can reject the null hypothesis, which asserts that the accuracy is solely an outcome of chance, if the model works well on a test set of images that it has not seen before.In conclusion, the alternate hypothesis in a CNN for facial expression recognition suggests that the model has learned to generalize from the training data to new images and is capable of correctly classifying facial expressions with a level of statistical accuracy that is not linked to chance.

To test the hypothesis that the output of a certain CNN layer is different for images of human faces compares to images of different types of facial expression , we would collect a sample of human face images from corresponding dataset, feed them into the CNN layer and compare the output using a statistical test such as a t-test or ANOVA. Based on that, we can conclude the result of this testing.

**Results** : Result of this test are following :

- Case (i) : From the t-test or ANOVA we can able to Accept null hypothesis if there is no significance difference between the distribution of feature extracted from the dataset and reference set of face images. That implies the population mean average face is nearly equal to the hypnotized mean average face.
- Case (ii) : From the t-test or ANOVA we can able to Accept alternative hypothesis if there is no significance difference between the distribution of feature extracted from the dataset and reference set of face images. Which giving us a scope to improve the accuracy for higher prediction.

**Conclusion of hypothesis testing:** Conlusion of this test are following :

- scenario 1: If we consider case(i) , then our model 2 is a suitable approach for this. In that case our accuracy score is nearby 7.0 which is maximum score with better accuracy prediction. So in this case Improving accuracy is not that much fruitful based on the result which we already have , but if we want to improve prediction score then we have to concentrate more on data pre-processing instead of convolution neural network.
- scenario 2: If we consider case(ii), in that case we will try to build a sequential convolution neural network which help us to produce more accurate prediction. The alternate hypothesis in CNNs for face recognition might state that the features learned by the CNN are able to capture unique expressions of each face, and that these features which are expressions can be used to distinguish between different individuals with high accuracy.

So, we saw that model 2 is suitable for scenario-1. Now, let’s explore scenario-2 and try to build a new model with improved accuracy score.

#### Building the architecture for model-3 

Using CNN how to solve significant difference between the distributions of features extracted from different faces, and that this difference can be used to accurately classify or identify different individuals. One solution is to use transferable learning to address the problem of significant variations between the distributions of features extracted from various faces and to correctly classify or identify various people using CNNs. In order to extract features from images, a pre-trained CNN is used on a sizable dataset like ImageNet. The network is then fine-tuned on a smaller dataset of pictures of faces. In this model we know that There is a significant difference between the distributions of features extracted from different faces, and that this difference can be used to accurately classify or identify different individuals.

**The required steps for modeling the architecture :**

- **deal with pre-trained model:** Replace the pre-trained model’s completely connected layers with new fully connected layers that are specifically designed for the face classification task. The proper number of neurons for the number of classes in your face dataset should be present in these new layers.
- ** Add new layers :** To modify the pre-trained model for the new dataset or job, add new layers. One or more fully connected layers that map the output of the convolutional layers to the number of classes in the new dataset are usually included in these new layers for face recognition.
- **Train the new layers:** While maintaining the weights of the base layers constant, train the new layers on the new dataset. As a result, the base layers’ earlier learned features are going to be preserved while the new layers may acquire features particular to the new dataset.
- **Fine-tune the entire model:** After training the new layers, continue training on the new dataset while unfreezing some of the base layers to fine-tune the entire model. For it to better successfully modify to the new dataset, the algorithm is going to be able to update its foundational weights as a result of this. Fine-tune the model on our face expression dataset by training it on the face images and their corresponding labels which are angry, disgust, Fear, Happiness, sadness, Neutral, Surprise.
- **Batch size:** The batch size determines how many images are processed at a time during each training iteration. Better convergence and generalization may result to batches that are smaller, but the training length may also rise. Larger quantities might speed up training, yet they could result in over-fitting. The batch size for this CNN model is 499.
- **Epochs:** The complexity of the model, the size of the training dataset, and the rate of convergence during training may all influence the number of epochs is needed to build a convolutional neural network (CNN) for face expression identification. In order to achieve high accuracy, it is normal to train the model for many epochs during the course of a few hours to a few days when training a CNN for expression identification. We take 30 epochs to learn our model architecture with maximum convergence state.

Here, model 3 architecture which represents all the convolutional layers, hidden layers and total no of parameter extractions in each step-by-step process.

**Fig. 29:**
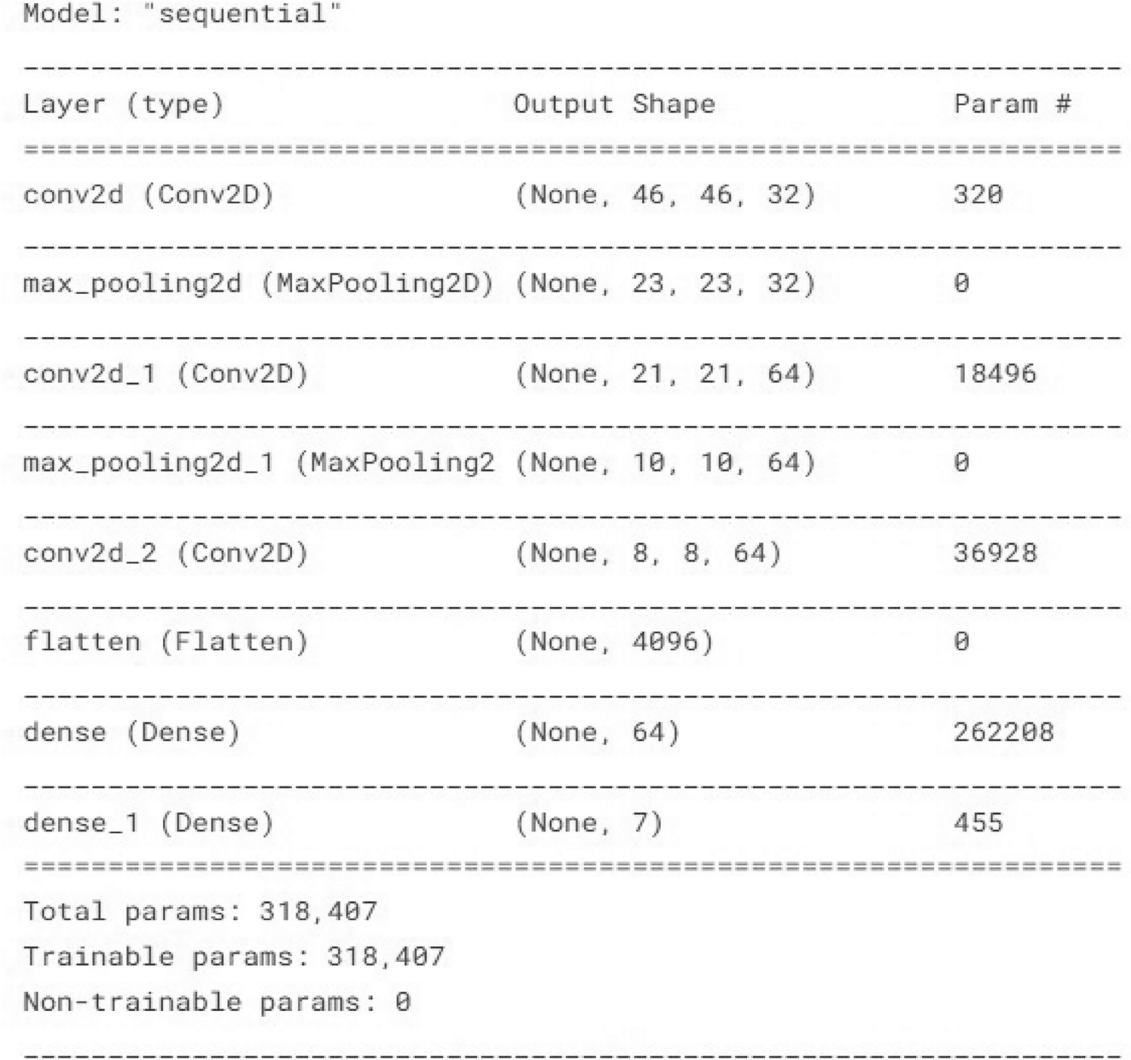
Artitecture of Convolution neural layers with parameter estimation for model-3.

In this Fig. 29, we get total 318,407 parameter estimations and total 318,407 trainable parameters estimation on the other hand we have 0 non-trainable parameters. The model has a sequential structure, with multiple convolutional and max-pooling layers followed by dense layers. Here’s the analysis:

- **Convolutional Layers (Conv2D):**The model starts with a Conv2D layer with 32 filters, each of size 3*x*3, resulting in an output shape of (46, 46, 32). The parameters (320) are a result of these filters plus the biases. The subsequent Conv2D layers progressively increase the number of filters to 64 (for the second and third Conv2D layers). This helps the network learn more complex and detailed features as the depth of the network increases.
  After each convolutional block, there is a MaxPooling2D layer that reduces the spatial dimensions by half. This down-sampling helps reduce the computational load and the number of parameters, while still retaining important features. The output shapes progressively reduce from (46, 46) to (23, 23), (21, 21) to (10, 10), and finally (8, 8) as we move deeper into the network.The third convolutional block (conv2d) still has 64 filters but works on smaller spatial dimensions (8, 8). It contains 36,928 parameters, which is higher than previous layers, indicating the network is learning more complex features here.
- **Flatten Layer and Dense Layers:** This layer flattens the 3D output of the previous convolutional layers into a 1D vector of 4,096 features. It acts as a bridge between the convolutional layers and the fully connected (dense) layers, preparing the data for classification.The first Dense layer has 64 units and 262,208 parameters. The number of parameters in a dense layer is: $\text {Parameters} = (\text {Input Units} \times \text {Output Units}) + \text {Output Units}$. This is where most of the learning happens, as it combines all the extracted features from previous layers and tries to establish relationships for classification. The final Dense layer has 7 units, corresponding to the number of classes (e.g., 7 facial expressions), with 455 parameters.
- **Parameter Concentration:** he majority of the parameters are concentrated in the dense layers (especially the first dense layer with 262,208 parameters). This is common in CNNs because fully connected layers often have the most parameters as they learn complex relationships. The convolutional layers have a significant number of parameters, but they increase progressively as the network depth increases, indicating that the network is learning increasingly abstract features.
- **Model Complexity:** The model is designed to start with fewer filters (32) in the first layer to capture basic features like edges and textures. As we go deeper, the number of filters increases (64) to capture more complex patterns. The model does not go as deep as some advanced architectures (e.g., ResNet), which might have hundreds of filters in deeper layers. However, it is still a well-structured model for learning relevant features for facial expression recognition. The number of parameters in a convolutional layer can be calculated using the formula:
  $$\begin{aligned} {\text {Parameters} = \left( K_h \times K_w \times C_{in} + 1 \right) \times C_{out}} \end{aligned}$$
  where,
  - $K_h$ and $K_w$ are the height and width of the filter/kernel.
  - $C_{in}$ is the number of input channels.
  - $C_{out}$ is the number of output channels (filters).
  - The “+1” accounts for the bias term for each filter.
  This sequential neural network performance is evaluated by the confusion matrix and using bar plot we will plot predictive class bar plot with actual class bar plot to compare the model prediction performance. To evaluate model performance, we use confusion matrix to shows number of correct and incorrect predictions made by our model. A confusion matrix can be used to assess how well a convolutional neural network (CNN) recognizes face expressions. A table that lists the number of accurate and inaccurate predictions a model made in relation to the real labels is called a confusion matrix. The real labels are denoted by the rows in the confusion matrix, while the predicted labels are represented by the columns. The number of times the predicted label matches the real label is displayed in each matrix cell.

#### Model-3 performance evaluation and visualization 

**Fig. 30:**
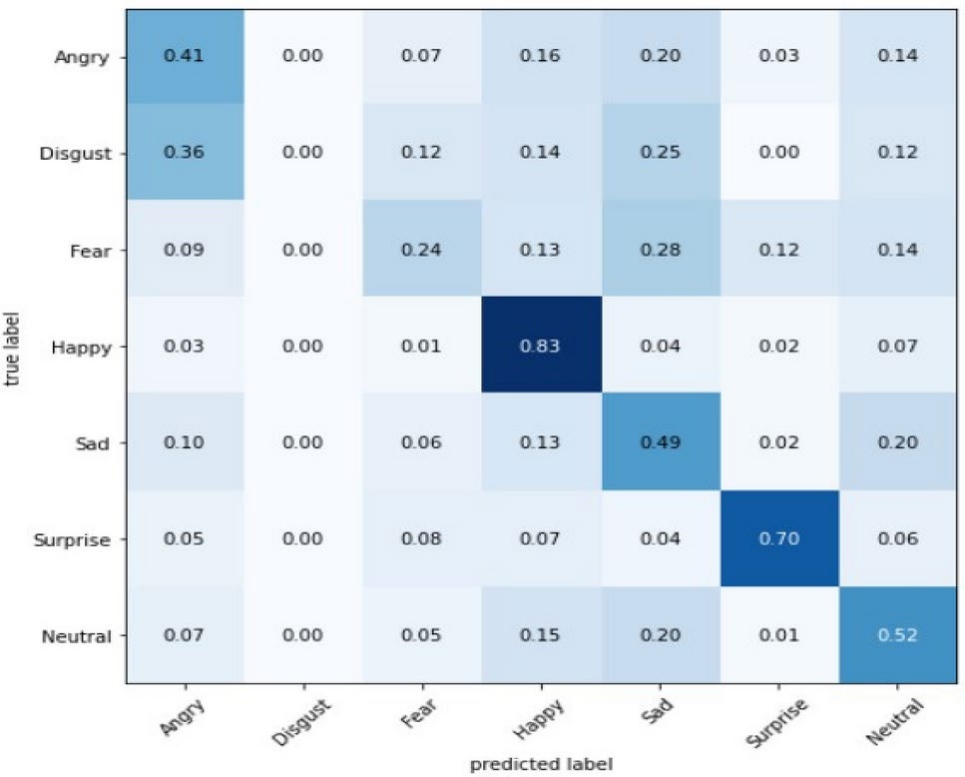
Confusion Matrix for model 3.

The following Fig. 30 is a confusion matrix for a model 2 of facial emotion recognition with 7 classes:

By examining the following metrics, accuracy, precision, recall, and F1-score, one can determine the model’s success based on the confusion matrix. The percentage of accurate forecasts over all predictions is how accuracy is calculated. Out of all positive predictions, precision can be defined as the proportion of true positives (positive examples that were accurately predicted). Confusion Matrix Insights:

- **Angry:** The model correctly identifies 41% of “Angry” expressions. However, it confuses “Angry” with “Disgust” (36%) and “Sad” (20%). This indicates that these expressions share similar features, making them difficult for the model to distinguish.
- **Disgust:**The model struggles significantly with “Disgust,” primarily predicting it as “Angry” (36%) or “Fear” (24%). This suggests that the model’s feature extraction process overlaps these emotions, which have intense and similar visual characteristics.
- **Fear: **Only 13% of “Fear” expressions are correctly classified. The Sequential CNN frequently mislabels them as “Disgust” (24%), “Sad” (28%), or “Surprise” (12%). The overlap in these misclassifications suggests that the model requires further refinement to differentiate these nuanced expressions.
- **Happy:** The model performs best with “Happy” expressions, achieving 83% accuracy. This indicates that the features representing “Happy” are distinct and well-learned by the Sequential CNN, making it the most accurately classified expression.
- **Sad: **For “Sad” expressions, the model achieves 49% accuracy. Common misclassifications include “Fear” (13%) and “Angry” (10%), indicating partial confusion with other negative emotions.
- **Surprise**The model shows a strong performance for “Surprise,” with a 70% accuracy rate. However, errors occur when it misclassifies “Surprise” as “Fear” (8%) or “Happy” (7%). These misclassifications suggest shared visual characteristics between these expressions.
- **Neutral:**The model shows moderate success for “Neutral” expressions, with 52% of instances correctly identified. Misclassifications as “Sad” (15%) or “Happy” (10%) indicate that subtle differences between these expressions may be challenging for the model to capture.

In conclusion, the Sequential CNN model shows strong performance, particularly in distinguishing “Happy” and “Surprise” expressions. However, further adjustments and enhancements are needed to improve accuracy for other, more similar facial expressions.

Recall quantifies the percentage of real positive experiences among all positive examples. The harmonic mean of recall and accuracy is referred to as the F1-score. High precision, recall, and F1-score characteristics should be present in a face expression recognition model. The precise values that are regarded as good, however, may change based on the application and dataset. An accurate performance is generally indicated by a confusion matrix with high values along the diagonal (true positives) and low values in the off-diagonal components (false positives and false negatives). If the model performs poorly, the model design, data pre-processing, or hyper-parameters may need to be changed in order to increase the model’s accuracy. Next, we use bar plot to show test labels with respect to all 7 types of different facial expression which is compared with prediction labels. The efficacy of a facial expression recognition model using conventional neural networks can be evaluated by comparing the distribution of predicted facial expressions. We can examine the distributions between the actual and predicted labels by generating histograms or density plots for each predicted emotion label. The two distributions should ideally be very similar, showing that the model accurately recognizes the facial expressions. The algorithm may not be functioning well and may need further optimization if the distributions are very dissimilar.

**Fig. 31:**
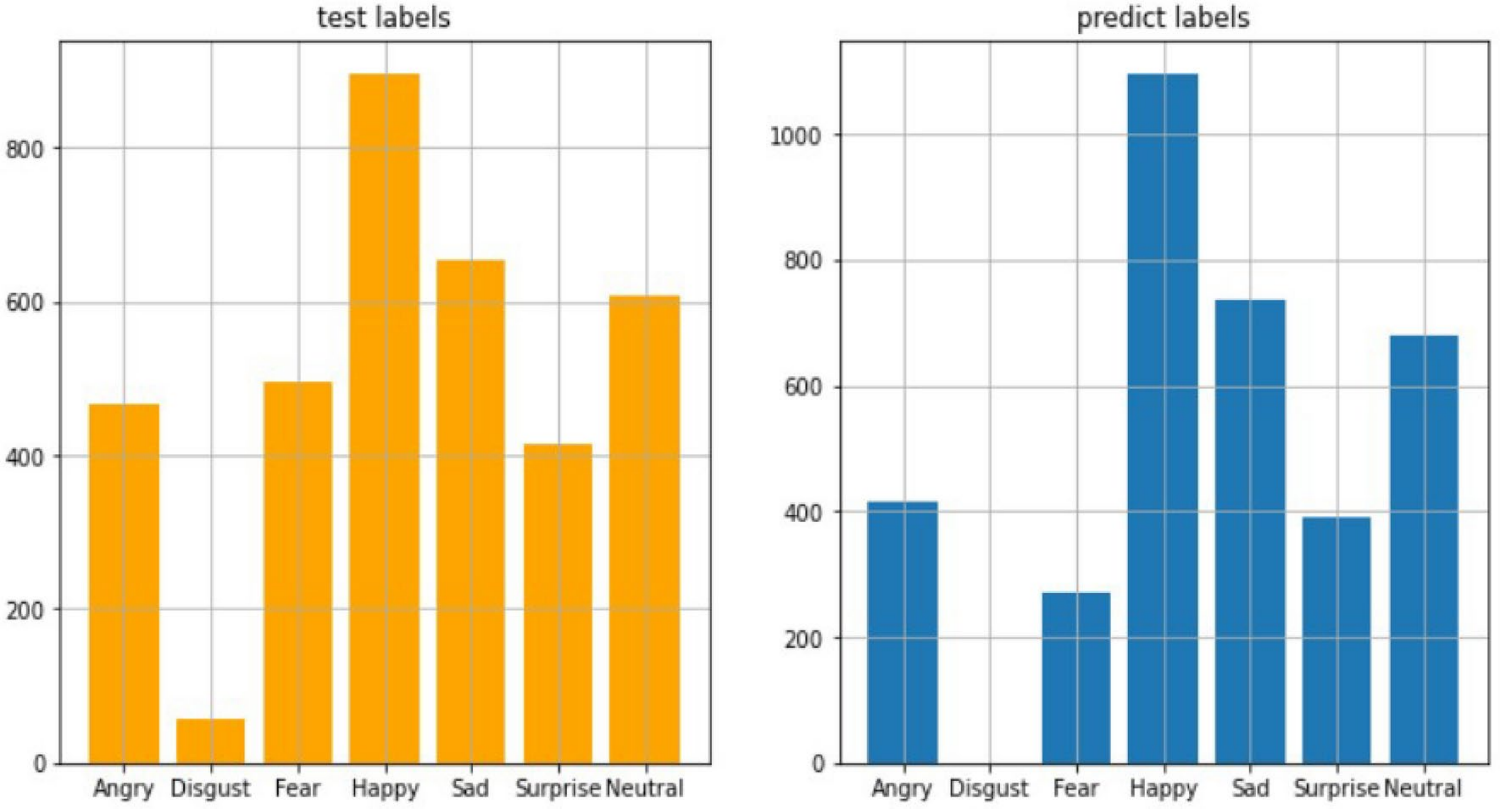
Plot Compare Distributions.

At last, we conclude the result for this model result with accuracy plot and model loss plot. Two important visualizations are in Fig. 31 that can aid in assessing how well a face expression recognition model built with conventional neural networks performs are the accuracy plot and model loss plot.

**Fig. 32:**
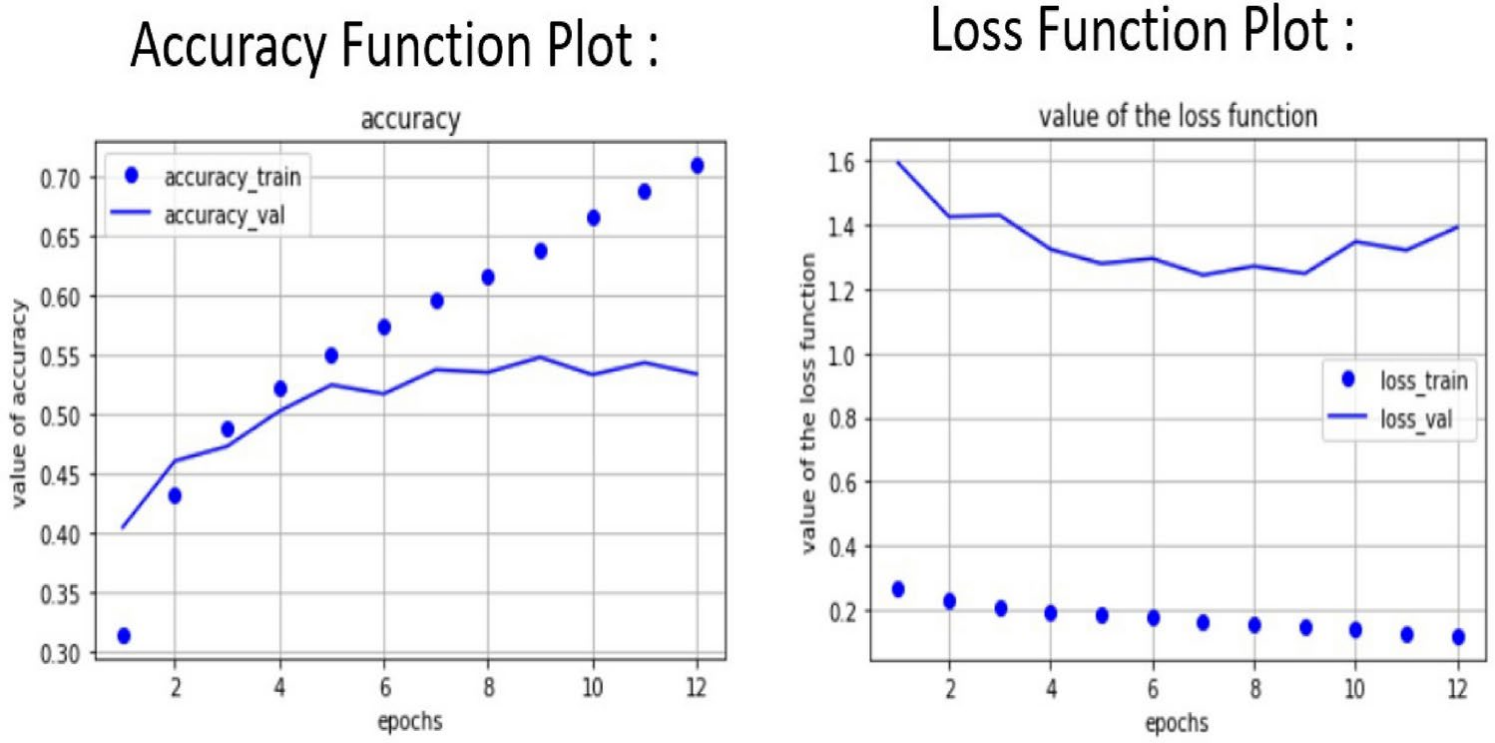
Visualization of entire dataset.

The accuracy plot displays the evolution of the model’s precision during training and validation. The accuracy should ideally rise for both the training and evaluation sets of data. However, if the validation data’s precision is noticeably lower than the training data’s, it might be a sign that the model is over-fitting. On the other hand, it may represent a sign that the model is not learning effectively if the training data’s precision is not increasing. The visualization for the accuracy plot and loss function plot is represented in Fig. 32 as

the accuracy for both training and validation is calculated as follows:

$$\begin{aligned} \text {Accuracy (epoch)} = \frac{\text {Correct Predictions}}{\text {Total Samples}} \end{aligned}$$

The accuracy function plot displays the training accuracy (accuracy_train) and validation accuracy (accuracy_val) over 12 epochs. Initial Epochs (1-2): the training loss starts high at around 16, indicating high prediction errors as the model is still adjusting its parameters. The validation loss is high, roughly matching the training loss, indicating that the model initially struggles to generalize. Middle Epochs (4-6): raining loss drops significantly to around 7, showing that the model is effectively minimizing error as it learns patterns from the dataset. Validation loss follows the downward trend but remains higher, approximately at 9, which suggests that the model’s ability to generalize is improving but still has room for refinement. Final Epochs (10-12) : training loss reduces furtherstabilizing around 0.2, indicating that the model has achieved a low error rate for the training data. The accuracy score for our model 3 is 0.7845 according to accuracy function plot and for validation accuracy score is 0.54712. For Loss function plot the loss score is 1.4 and the loss score for loss validation is 0.1002. The model loss plot shows how the model’s loss changes over the course of training and validation. Ideally, we want to see the loss decreasing for both the training and validation data. However, if the loss of the validation data is significantly higher than that of the training data, it could be an indication that the model is over-fitting. Conversely, if the loss of the training data is not decreasing, it could be an indication that the model is not learning well.

#### Output:

For output presentation we divide output in 2 parts where first part represent those bunch of photos which are classified in correct way with proper classification task. And the second part represented all the misclassified photos which causes model loss.

**Fig. 33:**
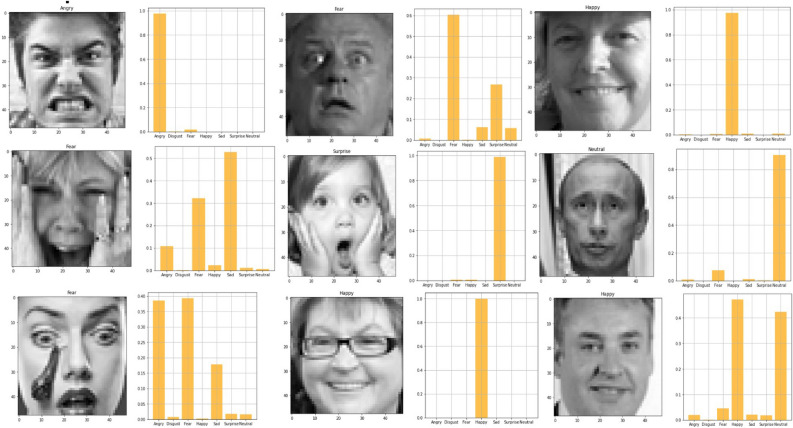
Well-classified Prediction facial image output.

**Output 1-Well-Classified Facial Images :** In the Fig. 33, a facial expression recognition model that uses traditional neural networks and produces a well-classified image output has successfully recognized the facial expression in the image. This suggests the image’s actual expression label fits the predicted expression label. Here, the visualization plot with 7 different type of attributes and attributes influencing behavior bar plot.

The model can properly classify facial expressions and has an extensive list of uses in psychology, human-computer interaction, and emotion recognition. Additionally, it can be used to keep track of emotions of individuals in an array of contexts, including security and monitoring. It is essential to remember that obtaining high accuracy in facial expression recognition is still a difficult job because of things like variations in facial expressions, lighting, and facial features. Therefore, to make sure the model is working well in all areas, the effectiveness of a facial expression recognition model should be evaluated using an array of metrics, including accuracy, precision, recall, and F1 score. Additionally, a diverse and accurate representation of the population under study should be included in the dataset used to train the model. To make sure that the model can recognize facial expressions correctly, the model architecture and training approach should also be thoughtfully selected and optimized.

**Fig. 34:**
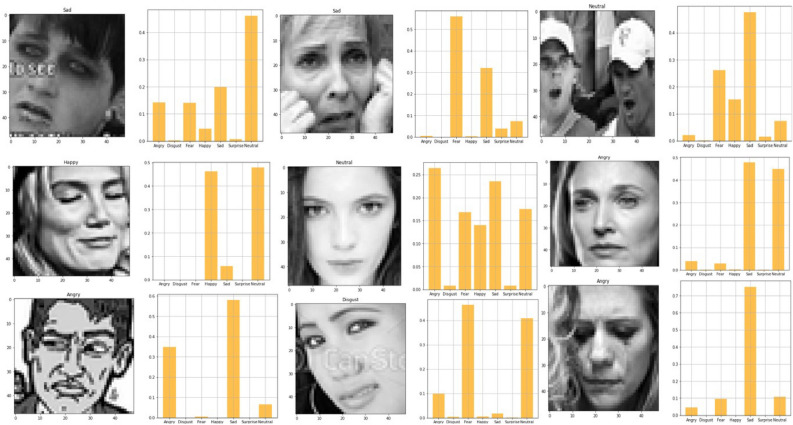
Miss-classified Prediction facial image output.

**Output 2-Miss-Classified Facial Images:** According to Fig. 34, an incorrectly recognized facial expression in a photograph is a sign that a facial expression recognition model using conventional neural networks misclassified the image. This indicates that the predicted expression label and the actual expression label for the picture are different. Mis-classifications can happen for a number of reasons, including differences in facial expressions, lighting, facial characteristics, and dataset bias. In situations like security and surveillance, a facial expression recognition model with a high rate of mis-classifications can have negative consequences.

The model can be improved to reduce mis-classifications by utilizing a larger and more varied dataset, adjusting the model architecture and training procedure, and adding extra features like facial landmarks or motion. The model’s effectiveness can be increased by employing additional strategies like data augmentation and transfer learning. In order to understand the model’s shortcomings, it is also crucial to assess the kinds of mis-classifications that the model is producing. For instance, if a certain facial expression or a particular demographic group is regularly misclassified by the model, there may be bias in the dataset or the design of the model. In conclusion, the occurrence of incorrectly classified picture outputs emphasize the necessity of continuous evaluation, monitoring, and advancement of facial expression recognition models in order to guarantee their accuracy and dependability in practical applications. The actual class for some of the images are not well classified which causes miss prediction of the class. As example, human facial expression is sad but it classified as angry, in that particular case our model is able to classify that image in sad category because of our neural network strategy. But it considers as misclassified because the actual class of that image is angry but our predictive class is sad which is correct with respect to that human facial expression. For this type reason we loss our accuracy score.

## Conclusion and future work

### Conclusion

This study examines how the neural network model learns to recognize facial emotions. It begins with Principal Component Analysis (PCA), which is essential for achieving higher accuracy, while PCA plots facilitate the analysis of feature combinations that hold significant information. The conclusions drawn from these findings are outlined below:

- Cross-validation (train-test split) was performed prior to implementing the classification task with the Support Vector Machine, which utilized pretrained pixel values to achieve an accuracy of 85% (with the actual class occurring 337 times). The micro average and weighted average were 0.83 and 0.86, respectively.
- Using Model 2, which is a densely connected convolutional neural network trained for approximately 30 epochs, we observed a loss value of 1.52 by the 30th epoch. A heatmap representation of the evolution matrix, comparing the training and validation sets, helped identify the true positive class between the two. In the training set, the model was able to correctly classify at least 3,000 images for each class. However, the maximum accuracy score achieved by Model 2 was 0.638936 at epoch 29 for training, indicating there is still room for improvement.
- We conducted a hypothetical analysis to assess the difference between the distribution of features extracted from the dataset and a reference set of face images. Based on this, we developed a sequential convolutional architecture with dense layers, consisting of 318,407 trainable parameters. Our model 3 achieved an accuracy score of 0.7845, as reflected in the accuracy plot. Additionally, each projected pixel image was depicted with a bar chart, illustrating individual prediction scores and the influence of different expressions on one another. This supports our conclusion that there is no significant difference between the feature distributions of the dataset and the reference face images.

### Future work

In the future, to enhance the performance of any facial recognition technique, it is crucial to carefully select a dataset that includes images with a minimum quality of 2KB. Additionally, curating a larger and more diverse dataset that captures a wide array of facial expressions across different demographics-such as age, ethnicity, and gender-will lead to better predictions for any architectural model used for facial recognition.

The next step in advancing this proposed work is to implement a combined approach using ResNet (Residual Networks) and LSTM (Long Short-Term Memory). In this approach, ResNet will act as a feature extractor to capture deep spatial features of the face, while the LSTM layer will learn the temporal sequences of these features in the data. This method is expected to outperform VGGNet because ResNet’s skip connections address the vanishing gradient problem, enabling the creation of deeper networks. This allows the model to learn more complex facial expression features without the risk of overfitting which can detect any type of possible human facial expressions and many more.

## Data Availability

The data that supports the findings of this study are available in https://drive.google.com/file/d/1_TBXHGTR-2U-2N9trHxBc0lLNVq1faky/view?usp=sharing.
